# KLF7 regulates super-enhancer-driven IGF2BP2 overexpression to promote the progression of head and neck squamous cell carcinoma

**DOI:** 10.1186/s13046-024-02996-y

**Published:** 2024-03-05

**Authors:** Hongshi Cai, Jianfeng Liang, Yaoqi Jiang, Ziyi Wang, Hongyu Li, Wenjin Wang, Cheng Wang, Jinsong Hou

**Affiliations:** 1 Department of Oral and Maxillofacial Surgery, Hospital of Stomatology, Guanghua School of Stomatology,, Sun Yat-Sen University, Guangzhou, 51055 China; 2 Guangdong Provincial Key Laboratory of Stomatology, Guanghua School of Stomatology, Sun Yat-Sen University, Guangzhou, 510080 China

## Abstract

**Background:**

Head and neck squamous carcinoma (HNSCC) is known for its high aggressiveness and susceptibility to cervical lymph node metastasis, which greatly contributes to its poor prognosis. During tumorigenesis, many types of cancer cells acquire oncogenic super-enhancers (SEs) that drive the overexpression of oncogenes, thereby maintaining malignant progression. This study aimed to identify and validate the role of oncogenic SE-associated genes in the malignant progression of HNSCC.

**Methods:**

We identified HNSCC cell-specific SE-associated genes through H3K27Ac ChIP-seq and overlapped them with HNSCC-associated genes obtained from The Cancer Genome Atlas (TCGA) dataset and Gene Expression Omnibus (GEO) datasets using weighted gene coexpression network analysis (WGCNA) to identify hub genes. The expression of IGF2BP2 and KLF7 in HNSCC was detected using clinical samples. To determine the biological role of IGF2BP2, we performed CCK-8, colony formation assay, Transwell migration assay, invasion assay, and orthotopic xenograft model experiments. Furthermore, we utilized a CRISPR/Cas9 gene-editing system, small-molecule inhibitors, ChIP-qPCR, and dual-luciferase reporter assays to investigate the molecular mechanisms of IGF2BP2 and its upstream transcription factors.

**Results:**

Our study identified IGF2BP2 as a hub SE-associated gene that exhibited aberrant expression in HNSCC tissues. Increased expression of IGF2BP2 was observed to be linked with malignant progression and unfavorable prognosis in HNSCC patients. Both in vitro and in vivo experiments confirmed that IGF2BP2 promotes the tumorigenicity and metastasis of HNSCC by promoting cell proliferation, migration, and invasion. Mechanistically, the IGF2BP2-SE region displayed enrichment for H3K27Ac, BRD4, and MED1, which led to the inhibition of IGF2BP2 transcription and expression through deactivation of the SE-associated transcriptional program. Additionally, KLF7 was found to induce the transcription of IGF2BP2 and directly bind to its promoter and SE regions. Moreover, the abundance of KLF7 exhibited a positive correlation with the abundance of IGF2BP2 in HNSCC. Patients with high expression of both KLF7 and IGF2BP2 showed poorer prognosis. Lastly, we demonstrated that the small molecule inhibitor JQ1, targeting BRD4, attenuated the proliferation and metastatic abilities of HNSCC cells.

**Conclusions:**

Our study reveals the critical role of IGF2BP2 overexpression mediated by SE and KLF7 in promoting HNSCC progression. Targeting SE-associated transcriptional programs may represent a potential therapeutic strategy in managing HNSCC.

**Supplementary Information:**

The online version contains supplementary material available at 10.1186/s13046-024-02996-y.

## Background

Head and neck cancers encompass various histologic types, with squamous cell carcinoma being the most common. Head and neck squamous cell carcinoma (HNSCC) is highly invasive and prone to metastasize cervical lymph nodes (CLN), leading to a higher risk of recurrence and metastasis, which is the primary cause of mortality [1, 2]. Despite advancements in diagnostic and therapeutic methods, the 5-year survival rate for HNSCC patients has not significantly improved in the last 30 years and remains around 50% [3, 4]. Therefore, it is crucial to comprehend the regulatory mechanisms governing invasive metastasis in HNSCC to develop innovative therapeutic strategies and improve the prognosis of patients.

Aberrant transcription of oncogenes driven by genetic and epigenetic alterations plays a critical role in the tumorigenicity and metastasis of cancers [5, 6]. Recent evidence suggests that super-enhancers (SEs) are important non-coding regulatory elements that determine the identity of different cell types. SEs enhance the expression of genes that are crucial for maintaining cellular identity during both organism development and disease progression [7–9]. SEs are DNA cis-regulatory elements that exhibit superior transcriptional activity, which are clusters composed of multiple neighboring enhancers. SEs are primarily composed of transcriptional activation-associated histone modifications (Histone 3 lysine 27 acetylation, H3K27Ac; Histone 3 lysine 4 monomethylation, H3K4me1), cofactors (bromodomain-containing protein 4, BRD4; mediator complex subunit 1, MED1), chromatin regulator (p300), RNA polymerase II (RNA Pol II), and cell type-specific transcription factors (TFs) by chromatin immunoprecipitation-sequencing (ChIP-seq) to define [9, 10]. Several studies have utilized the Ranking of Super-Enhancers (ROSE) algorithm to identify SEs in different types of cancers. These studies have confirmed that SEs are responsible for regulating the overexpression of important oncogenes (such as CCAT1, TP63, MYC, SOX2, KLF5, and FOSL1) during the development and progression of cancers. This regulation helps maintain the characteristics of cancer cells and promotes their malignant progression [11–17].

SEs are genomic regions that play crucial roles in controlling gene expression and cellular function. When TFs bind to SEs, BRD4 recognizes and attaches to acetylated histones, which in turn bind to the Mediator complex at the promoter region of the target gene. After transcription initiation, cyclin-dependent kinases 7 (CDK7)-containing TFIIH and CDK9-containing positive transcription elongation factor b (P-TEFb) are recruited by BRD4. These kinases phosphorylate the carboxy-terminal structural domain of RNA Pol II, facilitating the localization of RNA Pol II at the transcription start site and regulating gene transcription elongation [18, 19]. By disrupting SEs or inhibiting the SE-associated transcriptional program, it is possible to selectively inhibit the transcriptional activity of cancer cells driven by SEs [20–22]. Interestingly, Wu et al. discovered that there was less overlap of genes with altered expression by comparing the transcriptomic data of different types of cancer cells treated with JQ1, suggesting that, in different cellular environments inhibition of SE-associated transcription preferentially regulates vital cancer cell- or tissue-specific genes [23]. Currently, therapeutic strategies that directly target SEs by disrupting the SEs or inhibiting the SE-associated transcriptional program have shown promising results in various cancers [18, 20, 24].

In this study, we identified insulin-like growth factor 2 mRNA binding protein 2 (IGF2BP2) as a hub SE-associated gene in HNSCC. We observed a significant up-regulation of IGF2BP2 expression, which promotes tumorigenicity and metastasis in HNSCC. Mechanistically, we found that Krueppel-Like Factor 7 (KLF7) binds to the IGF2BP2-SE and drives its transcriptional activation and expression during the malignant progression of HNSCC. Finally, we demonstrated that targeting the SE-driven transcriptional program effectively reduced the tumorigenic potential and metastatic ability of HNSCC.

## Materials and methods

### Acquisition and analysis of ChIP-seq data

All H3K27Ac ChIP-seq data were obtained from the GEO database. The normal oropharyngeal mucosa H3K27Ac ChIP-seq data were obtained from the GSE112021 dataset [25]. The H3K27Ac ChIP-seq data of CAL27, Detroit562, and HN12 cells were obtained from the GSE128275 dataset [26]. The H3K27Ac ChIP-seq data of SCC25 cells were obtained from the GSE103554 dataset [27]. SRA files of the above ChIP-seq data were downloaded from the GEO database. After that, they were transformed into FASTQ format and subjected to quality control. The alignment of sequences with the human reference genome GRCh38 was performed using BWA. With the aid of MACS2, enriched regions within the ChIP-seq data were identified. SEs were further identified using ROSE with a stitching distance of 12.5 kb and visualized using the IGV Genome Browser. The SEs were assigned to genes with transcription start sites flanking the 50 kb window of the SEs. BEDGRAPH files from the GSE211473 dataset for UPCI-SCC-090, UM-SCC-104, and FaDu cells H3K27Ac ChIP-seq were converted to BIGWIG format and visualized on the IGV Genome Browser with the human reference genome hg19. HNSCC cell-specific SE-associated genes were further analyzed and enriched for disease gene networks using the 'DOSE' R package.

### Acquisition of high-throughput chromosome conformation capture (Hi-C) data

Hi-C data of UPCI-SCC-090, UM-SCC-104, and FaDu cells were obtained from the GSE211296 dataset. Subsequently, these data was visualized using the WashU Epigenome Browser (https://epigenomegateway.wustl.edu/browser/), with alignment to the human reference genome hg19.

### Acquisition and analysis of RNA-seq data

We obtained RNA-seq data and clinical data for TCGA-HNSCC from the UCSC Xena website (https://xenabrowser.net). Additionally, we downloaded gene expression matrices and clinical data from three other HNSCC datasets, namely GSE30784, GSE42743, and GSE41613, from the GEO database [28, 29]. To ensure uniformity, we converted the probe ID to gene symbols and extracted the mRNA transcriptome expression matrices. Subsequently, we integrated these matrices with the corresponding clinical data by utilizing R software. Our analysis required the transformation of the expression data of TCGA-HNSCC from count values to log2 (TPM + 1) values. To visually represent the data, we utilized the R packages 'ggpubr' and 'ggplot2' to generate boxplots. Additionally, we employed the 'survival' package for COX regression survival analysis and plotted the resulting survival curves using the 'survminer' package.

### Weighted gene coexpression network analysis (WGCNA)

To construct the weighted gene coexpression network, genes with a standard deviation (SD) > 1 were selected using the 'WGCNA' package [30]. Next, all samples were clustered to identify any missing values or outliers, and outlier samples were removed. The optimal soft threshold was then determined to ensure that the gene network followed a scale-free distribution. By converting the expression matrix into an adjacency matrix and further into a topological overlap matrix, we facilitated the hierarchical clustering of genes based on dissimilarity, leading to the formation of a clustering tree. The DynamicTreeCut algorithm was applied to partition the clustering tree into different modules, with the merging of modules that had a dissimilarity of less than 0.2. Lastly, we computed the correlation coefficient and *P*-value between each gene module and HNSCC. The gene module displaying the highest correlation coefficient and the smallest *P*-value was identified as the most pertinent gene module.

### Gene set enrichment analysis (GSEA)

Three HNSCC transcriptomes, TCGA-HNSCC, GSE30784, and GSE42743, were divided into high- and low-IGF2BP2 expression groups based on the median expression of IGF2BP2, respectively. We performed GSEA using the 'clusterProfiler' R package to compare the high- and low-IGF2BP2 expression groups. We assessed the enrichment of biological functions and pathways associated with IGF2BP2 expression using Hallmark and Kyoto Encyclopedia of Genomes (KEGG) gene sets.

### HNSCC specimens

Two sets of patients were used in this study: the HNSCC tissue microarray and the Sun Yat-sen University (SUSY) HNSCC patient cohort. The HNSCC tissue microarrays were acquired from two different sources, Shanghai Xinchao Biotechnology Co. (HOraC060PG01) and US Biomax (OR601c). The SUSY HNSCC patient group consisted of 109 HNSCC tissue specimens and 33 surgically removed adjuvant non-cancerous tissues (ANCTs) from the Department of Oral and Maxillofacial Surgery at the Hospital of Stomatology Sun Yat-sen University. Clinical and pathological information of the patients was gathered, and no preoperative treatment was given to any of the patients. Informed consent was obtained and the research was approved by the Medical Ethics Committee of the Hospital of Stomatology Sun Yat-sen University, following the guidelines set by the Declaration of Helsinki. The time from surgery until death from any cause or the last follow-up was considered as overall survival (OS), while the time from surgery until tumor recurrence (either local or distant) or the last follow-up was considered as disease-free survival (DFS).

### Immunohistochemistry (IHC)

All paraffin-embedded tissues were cut into 4.0-µm sections and underwent dewaxing and dehydration. Sodium citrate buffer was used for antigenic repair, after incubating the sections with 3% H2O2. Following that, the sections were blocked using anti-goat serum (#AR0009, BosterBio) and incubated overnight at 4◦C with primary antibodies against anti-IGF2BP2 (1:250, #11,601–1-AP, Proteintech, China), anti-KLF7 (1:300, #PA5-81,206, Thermo Fisher), anti-Pan-Keratin (1:1000, 4545S, Cell Signaling), or anti-BRD4 (1:200, #ab128874, Abcam). Subsequently, the sections were washed, incubated with a secondary antibody, and stained using the DAB detection Kit (GK600510, Gene Tech) with diaminobenzidine (DAB). Finally, the sections were re-stained with hematoxylin (D006, Nanjing Jiancheng Biotech). The IHC scores were calculated on a continuous scale of 0–300. This was achieved by multiplying the proportion of positive cells (ranging from 0 to 100%) by the intensity of staining, which was classified as 0 (no staining), 1 (weak), 2 (moderate), or 3 (strong). HNSCC tissues with IHC scores higher than 150 were classified as the high-expression group, while those with scores less than or equal to 150 were classified as the low-expression group.

### Cell culture and treatment

The present study utilized human HNSCC cell lines SCC25 and CAL27, along with human embryonic kidney-derived 293 T cells, which were procured from the American Type Culture Collection (ATCC). The SCC25 cells were cultivated in DMEM/F12, supplemented with 10% fetal bovine serum (FBS, #086–1500, WISENT) and 400 ng/mL hydrocortisone. On the other hand, CAL27 and 293 T cells were maintained in DMEM supplemented with 10% FBS, and all cells were incubated under constant conditions at 37 °C with 5% CO_2_. The small molecule inhibitors THZ1 (#V2557) and JQ1 (#V0411) were obtained from InvivoChem, whereas OTX-015 (#202,590–98-5) and CPI-637 (#1,884,712–47-3) were purchased from MedChemExpress. Upon reaching an approximate fusion rate of 60–70% overnight, the culture medium was replaced with varying concentrations of small molecule inhibitors for continuous cell culture and subsequent investigations.

### Cell transfection

To knock down IGF2BP2, we used short hairpin RNA (shRNA) targeting the IGF2BP2 gene, which was cloned into the pLKO.1 plasmid (Addgene). For overexpressing IGF2BP2 and KLF7, we cloned the full-length open reading frames (ORFs) of the human-derived IGF2BP2 and KLF7 into the pCDH-CMV-MCS-EF1-copGFP-T2A-Neo plasmid (System Biosciences, SBI) and pCDH-CMV- MCS-EF1-copGFP-T2A-Puro plasmid (System Biosciences, SBI), respectively. The KLF7 overexpression plasmid was tagged with the HA protein. To deplete IGF2BP2-SE, we designed CRISPR/Cas9 constructs with small guide RNA (sgRNA) targeting the IGF2BP2-SE region (E1, E2, and E3), which were cloned into the pU6-gRNA-Cas9-puro plasmid (Addgene). Lentiviruses were produced by transfecting the target plasmid with the packaging plasmids psPAX2 and pMD2.G (Addgene) into 293 T cells. SCC25 cells or CAL27 cells were incubated with the viral particles for 24 hours. After 48 hours of viral infection, cells were screened using puromycin (SCC25 cell: 1 mg/mL, CAL27 cell: 0.5 mg/mL) or G418 (SCC25 cell: 0.4 mg/mL, CAL27 cell: 0.2 mg/mL) medium for 1 week, and then the concentration was halved to continue screening the cells for 1 week. For siRNA transfection, HNSCC cells were grown overnight until reaching a fusion rate of approximately 50%. The appropriate negative control (si-NC) or small interfering RNA (siRNA) was added using the Pepmute Transfection Reagent (#SL100566, Signagen) according to the manufacturer's instructions. The sequences of the shRNA, sgRNA, and siRNA oligonucleotides are listed in Additional file 1: Table S1.

### Western blot

Cells were lysed with a mixture of RIPA buffer (#CW2333S, CWbio) and a protein inhibitor cocktail on ice for 15 minutes. After centrifugation at 12,500 rpm for 20 minutes at 4 °C, the supernatants were collected and assayed for protein concentration using the BCA Protein Assay Kit (#CW0014S, CWbio). Equal quantities of proteins were then separated by sodium dodecyl sulfate–polyacrylamide gel electrophoresis (SDS-PAGE; #PG112, Epizyme Biotech) and transferred to polyvinylidene fluoride (PVDF) membranes (#ISEQ00010, Millipore). The membranes were subsequently blocked with 5% bovine serum albumin at room temperature for 1 hour. Next, the primary antibodies were incubated at 4 °C overnight. The primary antibodies used in this study were anti-IGF2BP2 (1:1000, #14672S, Cell Signaling Technology), anti-β-ACTIN (1:1000, #19069S, Cell Signaling), anti-BRD4 (1:1000, #ab243862, Abcam), anti-MED1 (1:1000, #ab60950, Abcam), and anti-KLF7 (1:500, #SC398576, Santa Cruz Biotechnology). Following a 1-hour incubation period with the secondary antibodies, chemiluminescent images of the membranes were recorded accordingly.

### Quantitative real-time PCR (qRT-PCR)

According to the manufacturer's instructions, total RNA was isolated from the cells using RNAzol® RT (#RN190, Molecular Research Center). The Hifair III 1st Strand cDNA Synthesis kit (#11141ES60, Yeasen) was used to reverse-transcribe 1 μg of total RNA into complementary DNA. The qPCR reaction was performed using SYBR Green Master Mix (#11201ES08, Yeasen) on a LightCycler 480 II (Roche). Gene expression was calculated by 2^−ΔΔCt^ normalized to β-ACTIN. The primer sequences used for qRT-PCR are listed in Additional file 1: Table S2.

### Cell proliferation and colony formation assay

SCC25 and CAL27 cells were inoculated into 96-well plates overnight at a density of 2000 cells per well. Cell proliferation was measured at specific time points using the Cell Counting Kit-8 (CCK-8, #2003ES80, Yeasen). Following the provided, 100 µLof serum-free medium with 10% CCK-8 reagent was added to each well and the plates were incubated at 37 °C for 1 hour. The absorbance values were measured at 450 nm using a microplate reader (Biotek), and growth curves were plotted based on absorbance and time. For the cell colony formation assay, 500 SCC25 cells or 10,000 CAL27 cells were inoculated in 6-well plates and cultured for 7–10 days. Afterward, the cells were fixed using 4% paraformaldehyde and stained with 0.4% crystal violet. Direct counting was performed to determine the number of colonies in the SCC25 cells. The colony number for CAL27 cells involved the examination of three random fields under a microscope, magnified at 5 × .

### Cell migration and invasion assays

Serum-free medium was used to prepare SCC25 cell suspension at a density of 7.5 × 10^5^/mL and CAL27 cell suspension at a density of 6.0 × 10^5^/mL for conducting in vitro cell migration and invasion. After thorough mixing, 200µL of cell suspension was added to the upper chamber of the Transwell (for cell migration assay, 8 µm pore size, Corning) and Transwell coated with 0.33 mg/mL Matrigel (for cell invasion assay, 10 mg/mL, #354,234, Corning), while 800 µL of complete culture medium was added to the lower chamber. After incubation for 48 hours, 4% paraformaldehyde fixed the cells that passed through the cell chamber filter attached to the surface and stained with 0.4% crystal violet. To ensure comprehensive analysis, five independent fields of view were randomly selected for photographing and recording. The total number of cells in each field of view was then counted.

### Tumorigenesis and metastasis assay in vivo

Approval for animal experiments in this study was granted by the Institution Animal Care and Use Committee at Sun Yat-sen University. Female BALB/c-Nude mice, aged 4–6 weeks, were acquired from the Animal Experiment Center of the East Campus of Sun Yat-sen University and housed under pathogen-free conditions. To assess the influence of IGF2BP2 on tumor growth and metastasis in living organisms, the BALB/c-Nude mice were divided into three randomized groups: the control group, the shIGF2BP2 group, and the IGF2BP2 overexpression group, with 5 mice in each group. An orthotopic xenograft model was established by injecting 50μL suspension containing 1.0 × 10^6^ CAL27 cells into the tongue of anesthetized nude mice using a 27G insulin needle. Following cell inoculation, the nude mice were positioned in lateral recumbency with their tongues pulled out to prevent asphyxiation, and were exposed to a warming lamp to maintain body temperature. Mice with a weight loss exceeding 15% of their baseline were euthanized. After two weeks, the mice were anesthetized and euthanized by cervical dislocation. To investigate the effects of JQ1 treatment on tumor growth and metastasis in living organisms, nude mice were randomly assigned to two groups, each containing 6 mice, after intraperitoneal injection of 1.0 × 10^6^ CAL27 cells into their tongues to establish an orthotopic xenograft model for one week. The treatment group received a daily intraperitoneal injection of JQ1 at a dosage of 50 mg/kg, while the control group received only the vehicle for 14 days. After the experiment, the mice were euthanized by cervical dislocation following anesthesia. Tumor size was measured with vernier calipers, and tumor volume was determined using the formula: tumor volume = length × width × width/2. The tongues and CLNs of the nude mice were then collected, fixed, dehydrated, embedded, sectioned, and subjected to hematoxylin–eosin (H&E) staining and IHC staining.

### ChIP-qPCR

ChIP analysis was performed using the SimpleChIP Plus Enzymatic Chromatin IP Kit (Magnetic Beads) (#38191S, Cell Signaling) following the manufacturer's protocol. An approximate amount of 4 × 10^6^ cells was included in each ChIP reaction mixture. The cells underwent crosslinking with 1% formaldehyde at room temperature for 10 minutes, followed by glycine neutralization for 5 min. Subsequent steps involved resuspending the cells, digesting the chromatin with Micrococcal Nuclease, and sonication to obtain suitable DNA fragments. Dilution of the chromatin complexes occurred in ChIP dilution buffer, and immunoprecipitation was performed using these antibodies: anti-BRD4 (#ab243862, Abcam), anti-MED1 (#ab60950, Abcam), anti-H3K27Ac (#ab4729, Abcam), anti-normal rabbit IgG (#2729S, Cell Signaling), and anti-HA Tag (#66,006–2-lg, Proteintech). Elution of the chromatin from the antibody/protein G magnetic beads utilized ChIP elution buffer and was later transferred to a centrifugation column for DNA purification. Measurement of the immunoprecipitated DNA samples was done through qPCR. The resulting data were presented as a percentage of input DNA. For ChIP-qPCR primer sequences, please refer to  Additional file 1: Table S2.

### Dual-luciferase reporter assay

To validate the presence of KLF7 binding sites on the IGF2BP2-SE, we performed the cloning of a 322 bp segment that encompasses the wild-type (WT) KLF7 motif-binding sequence (obtained from chr3: 185,824,281–185,824,602) into the BglII site of the pmirGLO plasmid. This cloning process aimed to generate the luciferase reporter gene, designated as PmirGLO-WT. To create a mutated version of the KLF7 binding sites, the original sequence (GGGGCGGGG) was altered to AATAATTAT using the seamless cloning site-directed mutagenesis technique. The resultant plasmid was termed PmirGLO-mut. Following the plasmid construction, SCC25 and CAL27 cells were cultured in 12-well plates until they reached a fusion rate of approximately 60%. The cells were then transfected with pmirGLO, pmirGLO-WT, and pmirGLO-mut using the LipofectamineTM 3000 Transfection Kit (#L3000015, Thermo Fisher). After 48 hours of transfection, we measured the cellular luciferase activity using the Dual-Luciferase Reporter Gene Assay Kit (#11402ES60, Yeasen), following the manufacturer's instructions. To assess the translational efficiency of the reporter gene, the firefly luciferase activity was normalized with Renilla luciferase.

### Statistical analysis

Statistical analysis was carried out using GraphPad Prism 9.0 software (GraphPad Software, San Diego, CA, USA), and the data were presented as mean ± standard deviation (SD). To determine the normal distribution of the data, the Shapiro–Wilk test was performed. For comparing two groups, the student's t-test was employed, whereas for comparing three or more groups, ANOVA was used. The Log-Rank test and Kaplan–Meier method were utilized for survival analysis of clinical specimens. Additionally, Fisher's exact test was conducted to assess the differences in CLN metastasis in animal experiments. To examine the relationships between genes, Spearman's correlation was applied. A significance level of *P* < 0.05 was considered for all statistical analyses: *P < 0.05, **P < 0.01, ***P < 0.001.

## Results

### Screening of hub SE-associated gene IGF2BP2 in HNSCC

**Fig. 1:**
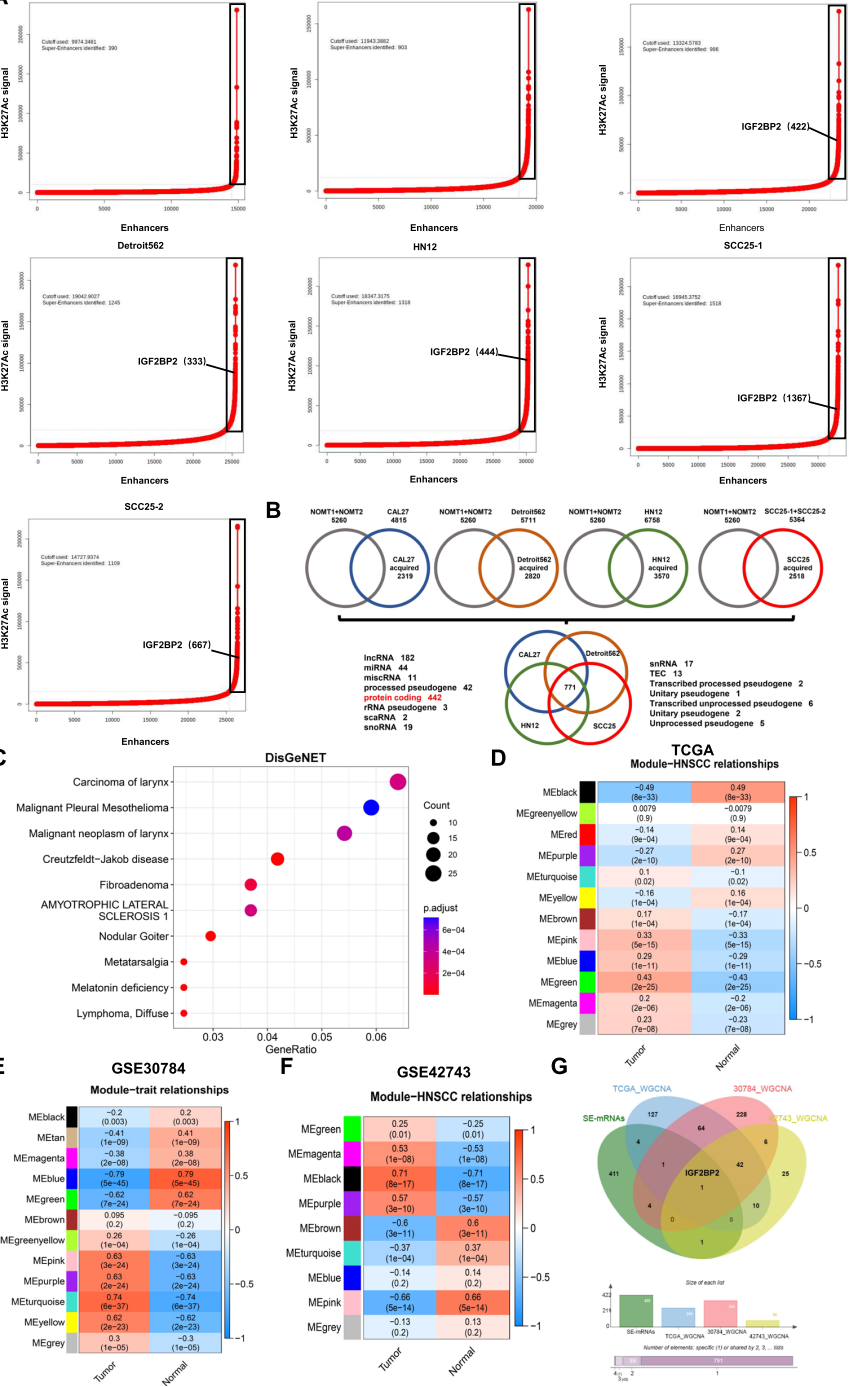
Screening of hub SE-associated gene IGF2BP2 in HNSCC. **A** The ROSE algorithm was utilized to plot the enhancer regions of normal oropharyngeal mucosa and HNSCC cell lines CAL27, Detroit562, HN12, and SCC25 in increasing order based on the H3K27Ac ChIP-seq signal, and enhancers above the inflection point of curve are defined as SEs. **B** In a comprehensive comparison of these HNSCC cell lines, 771 specific SE-associated genes and 442 specific SE-associated protein-encoding genes (mRNA) were identified, compared to SE-associated genes in normal oropharyngeal mucosa. **C** Disease-gene network enrichment analysis revealed that HNSCC cell-specific SE-associated genes were most enriched in carcinoma of the larynx. **D-F** WGCNA analysis identified the most relevant modules in the TCGA-HNSCC (**D**), GSE30784 (**E**), and GSE42743 (**F**) datasets for HNSCC, which were represented by the green (*r* = 0.47), turquoise (*r* = 0.74), and black (*r* = 0.71) modules, respectively. **G** Wayne diagram depicted the intersection of HNSCC cell-specific SE-associated protein-encoding genes with HNSCC-associated genes obtained from TCGA-HNSCC, GSE30784, and GSE42743 in WGCNA

Cancer cells commonly form oncogenic SEs on crucial oncogenes, which drive oncogene transcription and play a vital role in the initiation and maintenance of cancer characteristics. To explore the significance of SE alterations in the tumorigenesis of HNSCC, we compared the SE landscapes of two normal oropharyngeal mucosal tissues and four HNSCC cell lines (CAL27, Detroit562, HN12, and SCC25). Utilizing publicly available H3K27Ac ChIP-seq data, we employed the ROSE algorithm to recognize SEs and visualize curves. Enhancers located above the inflection point of the curve were defined as SEs. Multiple SEs were detected in both normal oropharyngeal mucosa and HNSCC cell lines (Fig. 1A, Additional file 2). By comparing the SE-associated genes between normal oropharyngeal mucosa tissues and HNSCC cells, we identified 2,319, 2,820, 3,570, and 2,518 specific SE-associated genes in the HNSCC cell lines CAL27, Detroit562, HN12, and SCC25, respectively. Additionally, we discovered 771 SE-associated genes that were specific to all four HNSCC cell lines, with 442 of them being protein-coding genes (Fig. 1B, Additional file 3). Analysis of the disease gene network indicated that HNSCC cell-specific SE-associated genes were primarily associated with a high number of cancers, particularly 'carcinoma of the larynx' (Fig. 1C). These findings suggest that HNSCC cells may acquire cis-regulatory SEs from oncogenes during the development of HNSCC.

To detect essential modules and genes linked to HNSCC, we generated WGCNA by using mRNA expression matrices obtained from three distinct HNSCC datasets: TCGA-HNSCC, GSE30784, and GSE42743. We used an optimal soft threshold of 0.85 to construct WGCNA based on scale-free topology (Additional file 4: Fig S1A). Modules with a similarity of less than 0.2 were merged using the DynamicTreeCut algorithm (Additional file 4: Fig S1B and C). By analyzing the correlation coefficients and *P*-values of each gene module in normal and HNSCC samples, we identified the most significantly correlated modules in each dataset. In the TCGA-HNSCC database, the green module containing 249 genes showed the strongest correlation with HNSCC (Fig. 1D, Additional file 5). In the GSE30784 dataset, the turquoise module containing 346 genes was most significantly correlated, while in the GSE42743 dataset, it was the black module containing 85 genes (Fig. 1E and F, Additional file 5). We then intersected 442 HNSCC cell-specific SE-associated protein-encoding genes with HNSCC-associated genes obtained from the three datasets in WGCNA. This analysis revealed that IGF2BP2 is the hub SE-associated gene in HNSCC (Fig. 1G).

### High expression of IGF2BP2 is correlated with clinicopathological variables and an unfavorable prognosis in HNSCC

**Fig. 2:**
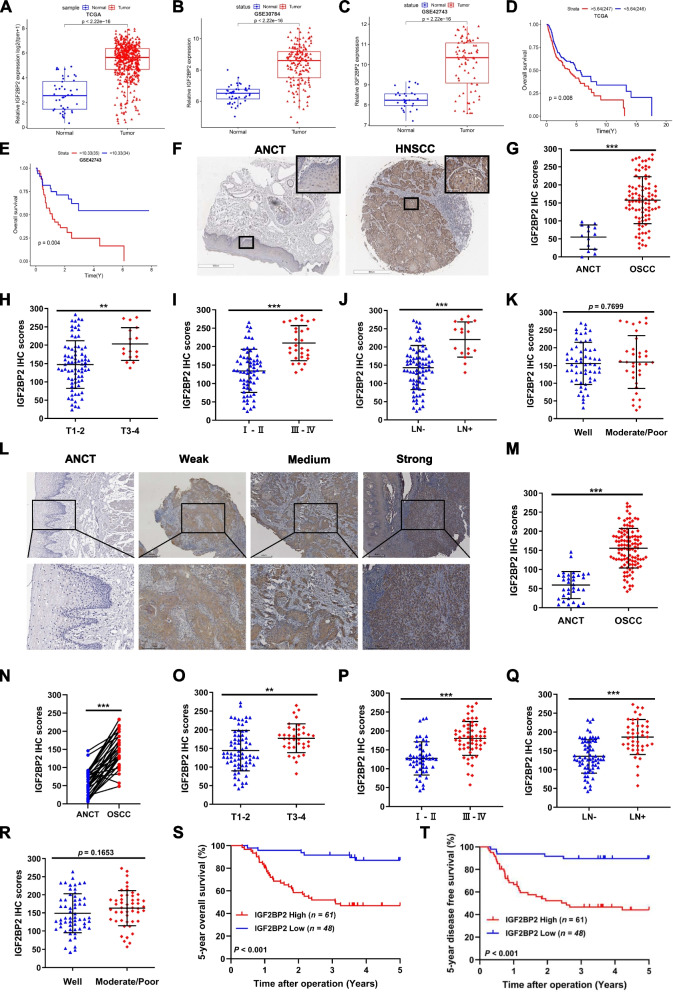
High expression of IGF2BP2 is correlated with clinicopathological variables and an unfavorable prognosis in HNSCC. **A-C** The analysis of TCGA-HNSCC (**A**), GSE30784 (**B**), and GSE42743 (**C**) datasets revealed an upregulation of IGF2BP2 mRNA expression in HNSCC. **D** and **E** Poor OS in patients with high IGF2BP2 mRNA expression in the TCGA-HSCCC (**D**) and GSE42743 (**E**) datasets. **F** Representative IHC staining images for IGF2BP2 in HNSCC tissue array. Scale bar of ANCT, 600 μm. Scale bar of HNSCC, 800 μm. **G** Histological scoring of IGF2BP2 in 94 HNSCC tissues and 14 ANCTs in HNSCC tissue arrays. **H–K** Histological scoring of IGF2BP2 in 94 HNSCC tissues with patients' T-stage (**H**), clinical stage (**I**), CLN metastasis (**J**), and pathological grade (**K**). **L** Representative IHC staining images for IGF2BP2 in HNSCC tissues. Scale bar of the upper panel, 500 μm. Scale bar of the lower panel, 250 μm. **M** Histological scoring of IGF2BP2 in 109 HNSCC tissues and 33 ANCTs. **N** Histological scoring of IGF2BP2 in 33 HNSCC tissue and their paired ANCTs. **O-R** Histological scoring of IGF2BP2 in 109 HNSCC tissues with patients' T-stage (**O**), clinical stage (**P**), cervical lymph node metastasis (**Q**), and pathological grade (**R**). **S** and **T** Kaplan–Meier survival curves of 5-year overall survival (**R**) and 5-year disease-free survival (**T**) based on patients with HNSCC with high- and low-expression IGF2BP2. *P < 0.05, **P < 0.01, ***P < 0.001. All the data are presented as mean ± SD from three independently performed experiments

SE-regulated genes show a specific and high level of expression in cancer cells and tissues. Initially, we examined the mRNA profiles of IGF2BP2 in three HNSCC datasets: TCGA-HNSCC, GSE30784, and GSE42743. The analysis revealed a significant increase in IGF2BP2 mRNA expression in HNSCC compared to normal tissues (Fig. 2A-C). Additionally, patients with elevated IGF2BP2 mRNA expression in these datasets exhibited poorer OS (Fig. 2D and E). To validate these findings, we assessed the protein expression of IGF2BP2 in HNSCC using tissue arrays composed of 94 HNSCC and 14 ANCTs. The results strongly supported the elevation of IGF2BP2 protein in HNSCC compared to ANCTs (Fig. 2F and G). Notably, the expression of IGF2BP2 protein positively correlated with tumor size (T stage), clinical stage, and CLN metastasis, but no significant difference was found in pathological grade (Fig. 2H-K). These results were consistent with those obtained from the SUSY HNSCC patient cohort (109 HNSCC and 33 ANCTs), where IGF2BP2 protein expression was upregulated in HNSCC and positively associated with tumor size, clinical stage, and CLN metastasis, without any statistical difference observed in pathological grade (Fig. 2L-R). Importantly, HNSCC patients with high IGF2BP2 expression experienced poor 5-year OS and 5-year DFS (Fig. 2S and T). These findings strongly suggest that IGF2BP2 is significantly upregulated in HNSCC and is closely linked to the malignant progression of the disease.

### IGF2BP2 promotes the proliferation, invasion, and metastasis of HNSCC

**Fig. 3:**
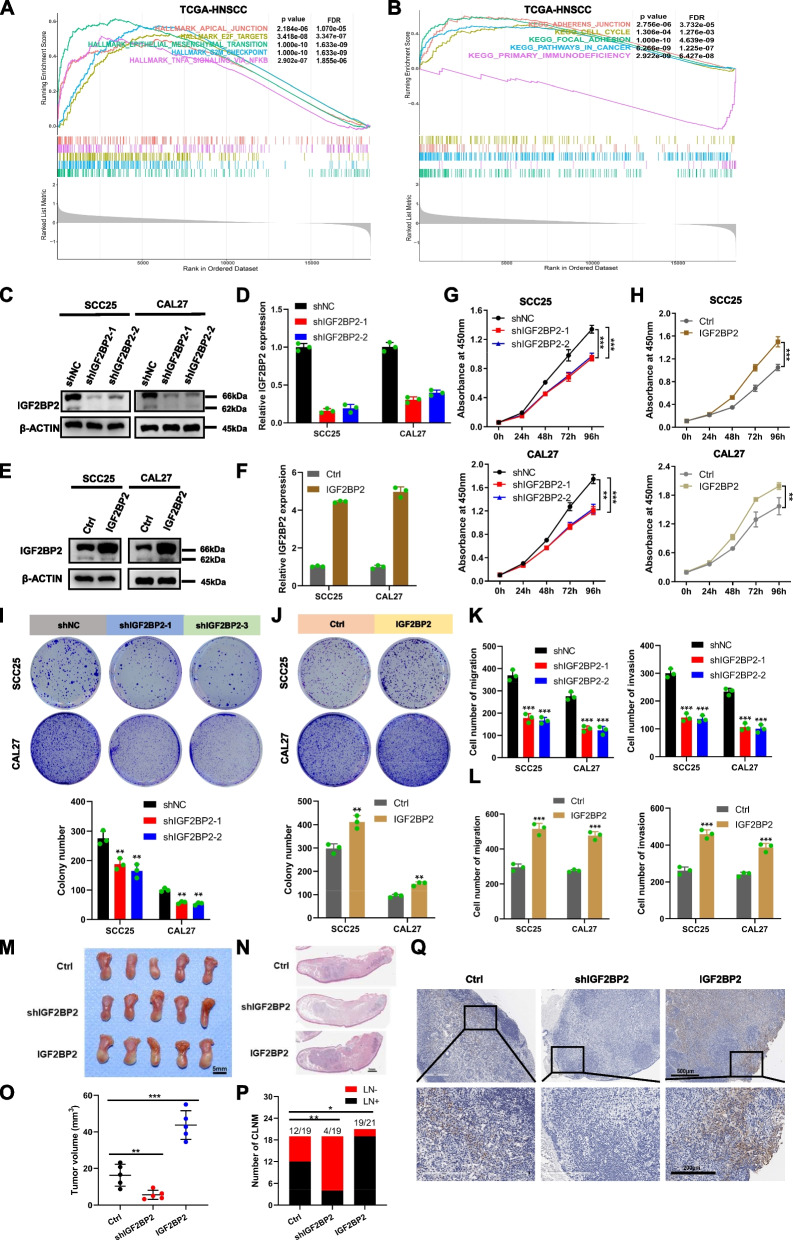
IGF2BP2 promotes the proliferation, invasion, and metastasis of HNSCC. **A** GSEA results showing functional enrichment of IGF2BP2 expression in Hallmark gene sets in the TCGA-HNSCC dataset. **B** GSEA results show functional enrichment of IGF2BP2 expression in KEGG gene sets in the TCGA-HNSCC dataset. **C** and **D** Western blot (**C**) and qRT-PCR (**D**) were used to detect the expression of IGF2BP2 after stable silencing in SCC25 and CAL27 cells. **E** and **F** The expression of IGF2BP2 after stable overexpression in SCC25 and CAL27 cells was detected by Western blot (**E**) and qRT-PCR (**F**). **G** and **H** The proliferation of SCC25 and CAL27 cells after stable silencing (**G**) or overexpression (**H**) of IGF2BP2 was detected using CCK-8 assay. **I** and **J** Clone formation assay was used to detect the clonal proliferation of SCC25 and CAL27 cells after stable silencing (**I**) or overexpression (**J**) of IGF2BP2. **K** The migration and invasion of IGF2BP2 knockdown SCC25 and CAL27 cells were measured. **L** The migration and invasion of IGF2BP2 overexpression SCC25 and CAL27 cells were measured. **M** Images of the tongue of BALB/C-Nude mice after inoculation with CAL27-Ctrl, CAL27-shIGF2BP2, and CAL27-IGF2BP2 cells, respectively. **N** Representative images of tumor tissues after H&E staining. Scale bar, 1 mm. **O** Comparison of tumor volumes after inoculation with CAL27-Ctrl, CAL27-shIGF2BP2, and CAL27-IGF2BP2 cells, respectively. **P** Fisher's exact test analysis of CLN metastasis percentage. **Q** Representative immunohistochemical staining images for Pan-Keratin in the CLN. Scale bar of the upper panel, 500 μm. Scale bar of the lower panel, 200 μm. *P < 0.05, **P < 0.01, ***P < 0.001. All the data are presented as mean ± SD from three independently performed experiments

To investigate the biological functions and pathways associated with the IGF2BP2 mRNA expression in HNSCC, we conducted a GSEA analysis on three datasets: TCGA-HNSCC, GSE30784, and GSE42743. In all three datasets, we observed enrichment of the Hallmark and KEGG gene sets in the high-expression group of IGF2BP2. These enriched gene sets were related to Epithelial-mesenchymal transition (EMT) and cell cycle, which are critical for the proliferation, invasion, and metastasis of HNSCC cells (Fig. 3A and B, Additional file 4: Fig S2, Additional file 6). Lentiviral-based SCC25 and CAL27 cells were then used to silence (shIGF2BP2-1 and shIGF2BP2-2) or overexpress IGF2BP2, and the transfection efficiency was confirmed through western blot and qRT-PCR (Fig. 3C-F). Silencing IGF2BP2 resulted in a decrease in the proliferative capacity of SCC25 and CAL27 cells, while overexpression of IGF2BP2 enhanced cell proliferation (Fig. 3G and H). Furthermore, silencing IGF2BP2 in SCC25 and CAL27 cells led to a significant reduction in colony formation in terms of both number and size compared to controls, whereas overexpression of IGF2BP2 increased clone formation (Fig. 3I and J). Additionally, silencing IGF2BP2 in SCC25 and CAL27 cells resulted in a decrease in migration and invasion ability, while overexpression of IGF2BP2 significantly enhanced cell migration and invasion (Fig. 3K and L, Additional file 4: Fig S3). Considering that the tongue is a primary site for HNSCC and is susceptible to CLN metastasis due to its frequent movement and abundant lymphatic blood vessels, we further conducted an in vivo orthotopic xenograft model using the tongue to examine the effects of IGF2BP2 on HNSCC growth and metastasis. According to the findings, it was observed that nude mice harboring CAL27-shIGF2BP2 cells exhibited diminished tumor sizes and volumes when compared to the control group. Conversely, nude mice carrying CAL27-IGF2BP2 overexpressing cells displayed enlarged tumors and tumor volumes (Fig. 3M-O). Additionally, through IHC staining, it was evident that the deletion of IGF2BP2 hindered CLN metastasis, while the overexpression of IGF2BP2 facilitated CLN metastasis (Fig. 3P and Q). To summarize, these discoveries provide conclusive evidence regarding the crucial role of IGF2BP2 in promoting the malignant progression of HNSCC.

### IGF2BP2 is a SE-driven gene

**Fig. 4:**
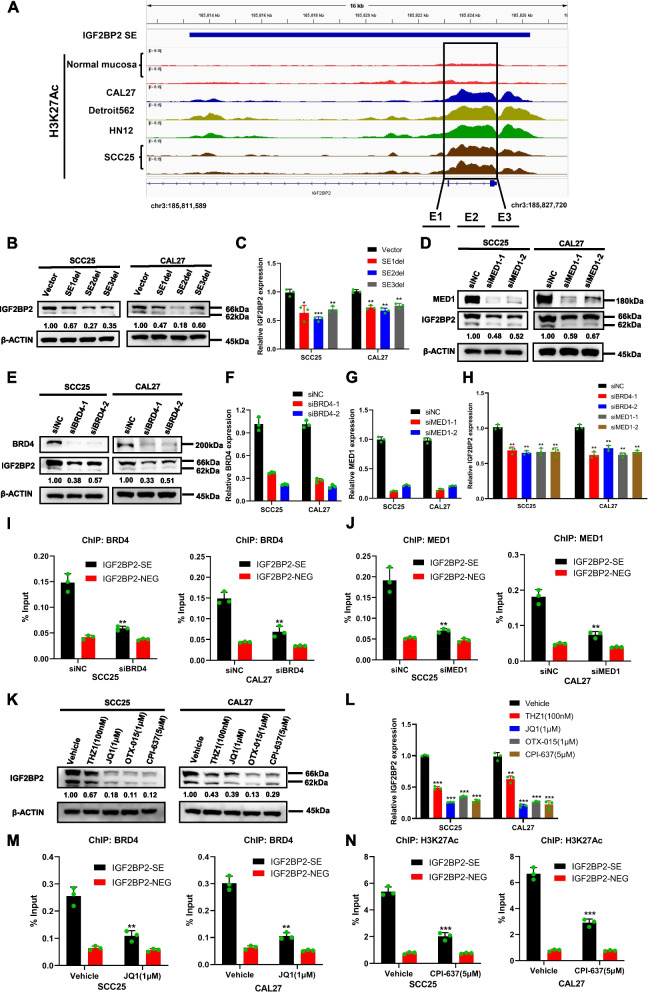
IGF2BP2 is a SE-driven gene. **A** Visualization of H3K27Ac level of IGF2BP2 with IGV Genome Browser. **B** and **C** Western blot (**B**) and qRT-PCR (**C**) were used to detect the expression of IGF2BP2 after the blockade of the IGF2BP2-SE region by CRISPR/Cas9 gene editing. **D** and **E** Western blot was used to detect the expression of IGF2BP2 after silencing BRD4 (**D**) or MED1 (**E**). **F–H** QRT-PCR was performed to assess the relative IGF2BP2 expression (**H**) after silencing BRD4 (**F**) or MED1 (**G**). **I** Silencing BRD4 reduced the BRD4 enrichment in the IGF2BP2-SE region. **J** Silencing MED1 reduced the MED1 enrichment in the IGF2BP2-SE region. **K** and **L** Detection of IGF2BP2 protein level and mRNA expression by Western blot (**K**) and qRT-PCR (**L**) after THZ1, JQ1, OTX-015, and CPI-637 treatment. **M** ChIP-qPCR showed that the enrichment of BRD4 in IGF2BP2-SE was reduced by treating with JQ1. **N** ChIP-qPCR showed that the enrichment of H3K27Ac in IGF2BP2-SE was reduced by treatment with CPI-637. *P < 0.05, **P < 0.01, ***P < 0.001. All the data are presented as mean ± SD from three independently performed experiments

We utilized the IGV Gene Browser to visualize the H3K27Ac ChIP-seq signals in the IGF2BP2 genome of HNSCC cell lines CAL27, SCC25, HN12, and Detroit562, which have approximately 13 kb of SE regions compared to normal oropharyngeal mucosa (Fig. 4A). According to our analysis of H3K27Ac ChIP-seq data from three HNSCC cell lines (UPCI-SCC-090, UM-SCC-104, and FaDu), we observed that H3K27Ac signals were significantly enriched in the IGF2BP2-SE region (Additional file 4: Fig S4A). Furthermore, when examining Hi-C data from public databases for UPCI-SCC-090, UM-SCC-104, and FaDu cells, we found strong evidence supporting a direct interaction between the SE region and the promoter region of IGF2BP2(Additional file 4: Fig S4B). We divided the non-promoter regions and the SE regions that are most enriched for H3K27Ac into three parts (E1, E2, and E3). Primer sequences were designed using these regions, and possible transcription factors were predicted (Fig. 4A). To disrupt the interactions between SE and promoter, CRISPR/Cas9 was utilized to precisely edit E1, E2, and E3. It is interesting to note that blocking the three individual constitutive sites on SE considerably diminished the levels of transcription and expression of IGF2BP2, thereby validating the positive regulation of IGF2BP2 expression by the SE in HNSCC cells (Fig. 4B and C).

In SE-associated transcriptional programs, the co-localization of BRD4 and MED1 is observed at chromatin sites where H3 is acetylated, specifically H3K27. Consequently, the modulation of BRD4 and MED1 expression also impacts the transcriptional activity of genes regulated by SEs [31]. To investigate this, we examined the effect of knocking down BRD4 or MED1 on IGF2BP2 expression in SCC25 and CAL27 cells. As depicted in Fig. 4D-H, both the mRNA and protein levels of IGF2BP2 were significantly decreased upon depletion of BRD4 or MED1. Importantly, we demonstrated that knockdown of BRD4 or MED1 resulted in decreased enrichment of BRD4 or MED1 within the IGF2BP2-SE region, respectively, consequently suppressing IGF2BP2 expression at both mRNA and protein levels (F ig. 4I and J).

CDK7, BRD4, and CBP/p300 are highly abundant in SEs and function together to activate transcriptional programs of SE-associated genes. Therefore, the expression of such genes is expected to be more vulnerable to inhibition of these transcriptional components. In our investigation, we targeted CDK7, BRD4, and CBP/p300 in CAL27 and SCC25 cells using small molecule inhibitors THZ1, JQ1, OTX-015, and CPI-637, respectively. Treatment with these inhibitors resulted in a notable decrease in mRNA and protein levels of IGF2BP2 (Fig. 4K and L). Moreover, we found that treatment with JQ1 or CPI-637 in SCC25 and CAL27 cells led to reduced occupancy of BRD4 and decreased H3K27Ac enrichment on the IGF2BP2-SE, respectively (Fig. 4M and N). These findings suggest that the IGF2BP2-SE region plays a critical role in driving the transcriptional program of IGF2BP2 and sustaining the malignant progression of HNSCC.

### BRD4 inhibition with JQ1 impairs HNSCC progression in vitro and in vivo

**Fig. 5:**
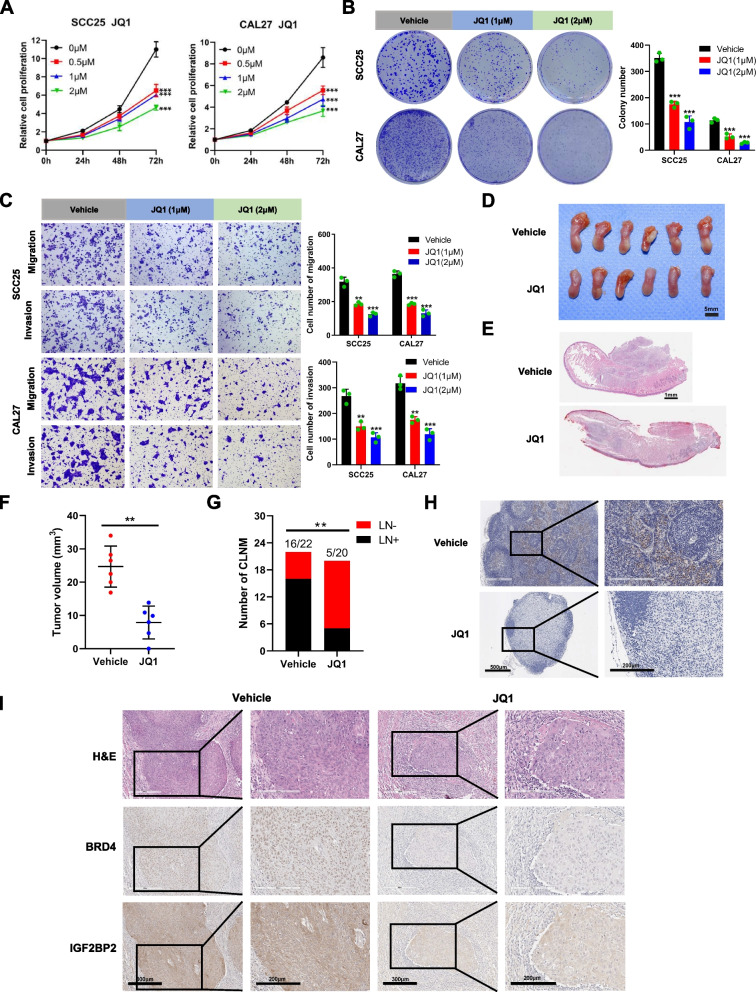
BRD4 inhibition with JQ1 impairs HNSCC progression in vitro and in vivo. **A** CCK-8 assay was used to detect the proliferation of SCC25 and CAL27 cells after treatment with JQ1. **B** Clone formation assay detected the clonal proliferation of SCC25 and CAL27 cells after treatment with JQ1. **C** The migration and invasion of SCC25 and CAL27 cells after treatment with JQ1 were photographed and measured. Scale bar, 100 μm. **D** Images of the tongue of BALB/c-Nude mice after injection of CAL27 cells and treatment with control vehicle or JQ1. **E** Representative images of tumor tissues after H&E staining. Scale bar, 1 mm. **F** Comparison of tumor volumes treated with control vehicle or JQ1. **G** Fisher's exact test analysis of the percentage of CLN metastasis. **H** Representative immunohistochemical staining images for Pan-Keratin in the CLN. Scale bar of the upper panel, 500 μm. Scale bar of the lower panel, 200 μm. **I** Representative images of H&E staining, BRD4 IHC staining, and IGF2BP2 IHC staining in HNSCC orthotopic xenografts treated with control vehicle or JQ1. Scale bar of the left panel, 300 μm. Scale bar of the right panel, 200 μm. *P < 0.05, **P < 0.01, ***P < 0.001. All the data are presented as mean ± SD from three independently performed experiments

JQ1, a commonly used inhibitor of BRD4 in preclinical research, hinders the binding of BRD4 to acetylated histones, thereby exerting anti-cancer effects both in vitro and in vivo. This is achieved by reducing BRD4 occupancy in the SEs region and decreasing the level of MED1 binding. Consequently, RNA Pol II transcription and elongation are arrested, inhibiting the excessive transcription of specific SE-associated oncogenes [32, 33]. To evaluate the therapeutic potential of JQ1 in HNSCC, we performed experiments to investigate its inhibitory effects on the growth of SCC25 and CAL27 cells. The results shown in Fig. 5A and B demonstrate that treatment with JQ1 significantly suppressed the proliferation and ability to form colonies in SCC25 and CAL27 cells. Likewise, JQ1 treatment inhibited the migration and invasion of HNSCC cells (Fig. 5C). To assess the anti-cancer effects of JQ1 in vivo, we established a tongue in an orthotopic xenograft model and treated nude mice with either JQ1 or vehicle. Compared to the group treated with vehicle, the tongue graft tumors in the JQ1-treated group exhibited smaller sizes. IHC staining revealed a reduced number of CLN metastasis in the JQ1-treated group, further demonstrating the anti-cancer effect of JQ1 (Fig. 5D-H). As expected, reduced expression of BRD4 and IGF2BP2 was also detected in orthotopic xenografts from the tongue of JQ1-treated mice compared with the group treated with vehicle (Fig. 5I).

### SE-associated IGF2BP2 is transcriptionally activated by KLF7

**Fig. 6:**
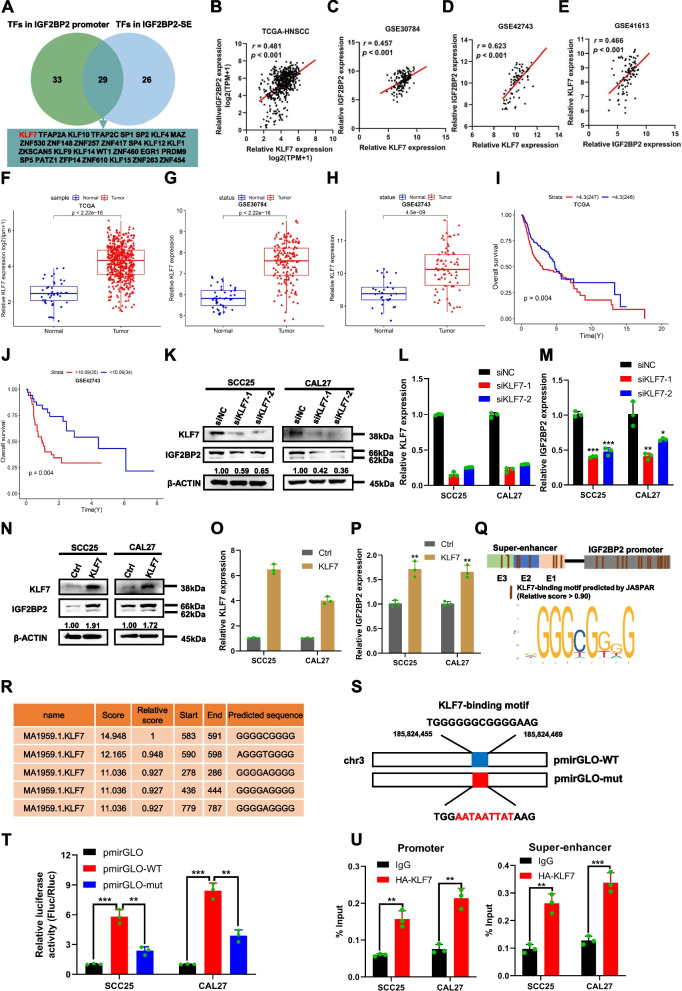
SE-associated IGF2BP2 is transcriptionally activated by KLF7. **A** Wayne diagram depicted the intersection of transcription factors bound to IGF2BP2-SE and promoter. **B-E** Correlation of IGF2BP2 expression with KLF7 in four HNSCC datasets, TCGA-HNSCC (**B**), GSE30784 (**C**), GSE42743 (**D**), and GSE41613 (**E**). **F–H** TCGA-HNSCC (**F**), GSE30784 (**G**), and GSE42743 (**H**) datasets revealed that KLF7 expression was upregulated in HNSCC. **I** and **J** Poor overall survival in patients with high KLF7 expression in the TCGA-HSCCC (**I**) and GSE42743 (**J**) datasets. **K** Western blot was used to assess the protein level of IGF2BP2 after silencing KLF7. **L** and **M** QRT-PCR detected the relative IGF2BP2 expression (**M**) after silencing KLF7 (**L**). **N** Western blot was performed to detect the protein level of IGF2BP2 after stable overexpression of KLF7. **O** and** P** QRT-PCR were used to assess the relative IGF2BP2 expression (**P**) after stable overexpression of KLF7 (**O**). **Q** Prediction of KLF7 binding sites and binding motifs in IGF2BP2-SE and promoter regions using the JASPAR website. **R** Binding sites and binding motifs of KLF7 in the SE region of the IGF2BP2 coding chain. **S** Schematic representation of wild-type and mutant KLF7 binding motifs in IGF2BP2-SE region. **T** Reduced luciferase activity after mutating the binding site of KLF7 to IGF2BP2-SE region. **U** The bindings of KLF7 to the IGF2BP2-SE and promoter regions were examined by ChIP-qPCR. *P < 0.05, **P < 0.01, ***P < 0.001. All the data are presented as mean ± SD from three independently performed experiments

SEs are DNA cis-regulatory elements that consist of TF binding sites. These binding sites are required to interact with TFs, forming SE-promoter loops that co-regulate the transcriptional program of genes. Alterations in TF enrichment can impact the activity of SEs, consequently affecting gene expression [34, 35]. To identify TFs capable of activating IGF2BP2-SE, we utilized the JASPAR database and predicted TFs that could potentially bind to both IGF2BP2-SE and promoter regions. We applied a filter (Score > 500, *P* < 10^–5^) and identified a total of 55 TFs that potentially bind within the IGF2BP2-SE region, with the most abundant H3K27Ac. Additionally, 62 TFs were found to bind in the promoter region (2 kb region upstream of the gene), and 29 of these TFs bound to both IGF2BP2-SE and the promoter region (Fig. 6A, Additional file 7). We further analyzed the expression correlation of IGF2BP2 mRNA with these 29 TFs using four HNSCC datasets, namely, TCGA-HNSCC, GSE30784, GSE42743, and GSE41613. Among these TFs, KLF7 showed the strongest positive correlation with IGF2BP2 (Fig. 6B-E, Additional file 1: Table S3). Moreover, in the HNSCC dataset, KLF7 mRNA expression was significantly upregulated in HNSCC tissues when compared to normal tissues (Fig. 6F-H). Furthermore, HNSCC patients with high KLF7 mRNA expression experienced unfavorable OS (Fig. 6I and J). Considering KLF7's established role as a transcriptional activator and its association with HNSCC progression, we hypothesized that it might bind to IGF2BP2-SE to regulate the transcription and expression of IGF2BP2. To validate this hypothesis, we knocked down the expression of KLF7 in SCC25 and CAL27 cells, which resulted in reduced levels of IGF2BP2 protein and mRNA expression (Fig. 6K and L). Conversely, the overexpression of KLF7 enhanced the transcription and expression of IGF2BP2 (Fig. 6N-O).

**Fig. 7:**
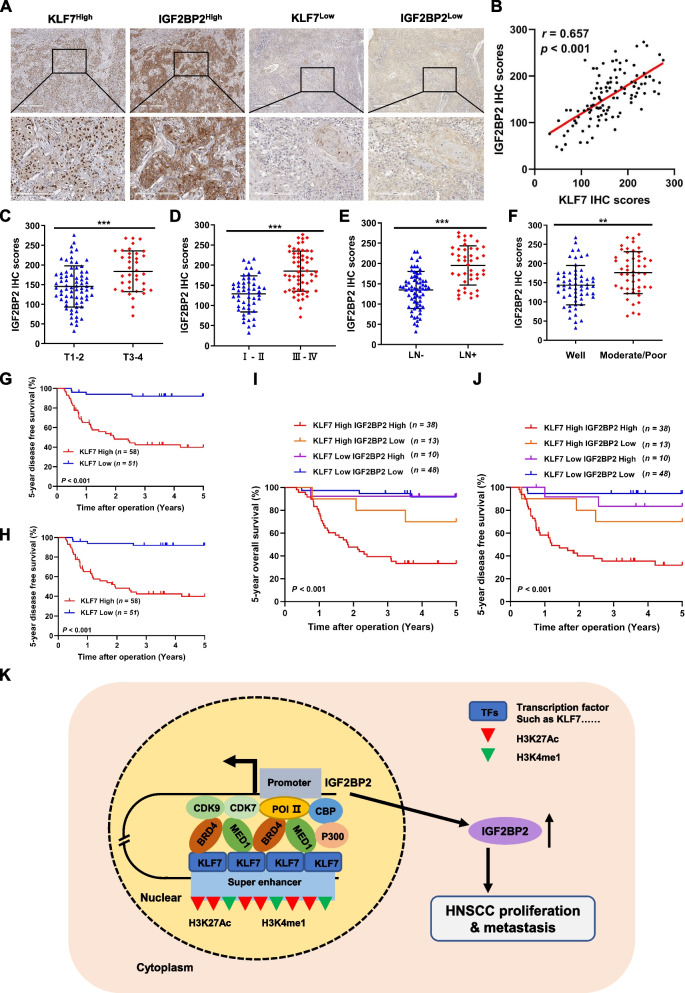
Correlation of KLF7 and IGF2BP2 expression in HNSCC and its clinical significance. **A** Representative immunohistochemical staining images of IGF2BP2 and KLF7 expression in HNSCC tissues. **B** Correlation analysis of IGF2BP2 and KLF7 expression in 109 HNSCC tissues; **C-F** Histological scoring of KLF7 in 109 HNSCC tissues with patients' T-stage (**C**), clinical stage (**D**), CLN metastasis (**E**), and pathological grade (**F**). **G** and **H** Kaplan–Meier survival curves of 5-year overall survival (**G**) and 5-year disease-free survival (**H**) based on patients with HNSCC with high and low KLF7 expression. **I** and **J** Kaplan–Meier survival curves of 5-year OS (**I**) and 5-year DFS (**J**) of patients in the high and low KLF7 expression groups combined with IGF2BP2. K Mechanistic model of KLF7-regulated SE-driven IGF2BP2 overexpression promoting malignant progression in HNSCC. *P < 0.05, **P < 0.01, ***P < 0.001. All the data are presented as mean ± SD from three independently performed experiments

In order to further validate KLF7's direct influence on the transcriptional expression of IGF2BP2 through SE, we conducted an analysis of the binding sites and motifs of KLF7 in both IGF2BP2-SE and its promoter, using the JASPAR database. We then selected the binding sites and motifs with the highest relative scores of KLF7 in IGF2BP2-SE (GGGGCGGGGGG) to generate the wild-type (PmirGLO-WT) and mutant (PmirGLO-mut) dual luciferase plasmids (Fig. 6Q-S). As a result, the luciferase activity of PmirGLO-WT transfected cells experienced a significant increase, while the luciferase activity of PmirGLO-mut transfected cells underwent a significant decrease (Fig. 6T). Our findings were further corroborated by ChIP-qPCR experiments, which confirmed the enrichment of KLF7 at both IGF2BP2-SE and its promoter (Fig. 6U). This supports our initial hypothesis that KLF7 plays a crucial role in promoting the activity of IGF2BP2-SE. Moving forward, to explore the relationship between KLF7, IGF2BP2, and the clinical implications in HNSCC, we examined the protein expression of KLF7 and IGF2BP2 in the SUSY HNSCC patient cohort using IHC staining. The protein levels of KLF7 exhibited a significant positive correlation with those of IGF2BP2 (Fig. 7A and B). Furthermore, high KLF7 expression was found to be positively correlated with advanced T stage, clinical stage, and CLN metastasis, as well as a lower degree of pathologic grade (Fig. 7C-F). It is worth noting that elevated KLF7 expression served as a predictor of poorer 5-year OS and 5-year DFS for HNSCC patients (Fig. 7G and H). Interestingly, patients with low expression of both KLF7 and IGF2BP2 exhibited the most favorable prognosis (Fig. 7I and J).

## Discussion

A thorough understanding of the intricate molecular mechanisms responsible for the invasive metastasis of HNSCC, a highly aggressive cancer prone to spreading, remains an unresolved challenge. Fundamental research has shown that oncogenes in several human cancers acquire cis-regulatory SEs throughout their malignant progression, thereby influencing their transcriptional expression [36–38]. Thus, it becomes paramount to investigate how SEs regulate crucial oncogenes, as it may yield new opportunities for therapeutic interventions and enhance the prognosis of HNSCC patients. Our investigation unraveled the association between HNSCC cell-specific SEs and multiple oncogenes, suggesting their acquisition during the development of HNSCC tumors. Furthermore, we unequivocally demonstrated that IGF2BP2, an essential protein-encoding gene associated with SEs, enhances the proliferation, invasion, and metastasis of HNSCC cells in both in vitro and in vivo experiments. The enrichment of BRD4, MED1, and H3K27Ac modification on the IGF2BP2-SE was found to synergistically activate its transcriptional program. Moreover, our findings indicate that the transcription factor KLF7 directly binds to the SE as well as the promoter regions of IGF2BP2, thereby enhancing its transcriptional activity and driving the expression of IGF2BP2 (Fig. 7K).

SEs are known to be specific to certain cells and tissues, and they play a crucial role in the overexpression of key oncogenes in cancer cells. This, in turn, contributes to the initiation and maintenance of tumor properties [39, 40]. For instance, in esophageal adenocarcinoma, SEs that are specific to this type of cancer regulate the overexpression of pro-carcinogenic transcription factors like ELF3, KLF5, GATA6, and EHF. These transcription factors activate the STAT3 and PI3K/AKT signaling pathways, thereby promoting cell proliferation [41]. Another study conducted on normal liver cells and hepatocellular carcinoma tissues revealed that cancer-specific SEs drive the overexpression of the oncogene SPHK1, leading to the proliferation and metastasis of hepatocellular carcinoma cells [36]. These findings emphasize the role of SEs in promoting the expression of oncogenes, activating pro-oncogenic signaling pathways, maintaining the identity of cancer cells, and facilitating the progression of malignancy. In a previous study, we also demonstrated the involvement of SE-associated FOSL1 in the tumorigenicity and metastasis of HNSCC by impacting stemness and EMT [17]. In our current research, we have identified IGF2BP2 as a gene associated with SEs specific to HNSCC cells, based on the analysis of H3K27Ac ChIP-seq and transcriptome data.

IGF2BP2 is situated on chromosome 3q27.2. It is a newly discovered N6-methyladenosine (m^6^A)-reading protein that is upregulated in various cancer types. Its association with cancer progression and its negative impact on cancer prognosis are well-established. The m^6^A-dependent manner in which IGF2BP2 operates enhances mRNA stabilization and translation. Furthermore, it plays a crucial role in regulating cell proliferation, metabolism, EMT, migration, and invasion [42–47]. Particularly in the context of HNSCC, IGF2BP2 acts as a facilitator for cell migration, invasion, and EMT by increasing the stability of Slug mRNA in an m^6^A-dependent manner [48]. Our investigation, combining data from public databases and analysis of clinical samples, has demonstrated a significant increase in IGF2BP2 expression in HNSCC. This increase is closely associated with metastasis and poor prognosis in patients. GSEA analysis suggests that IGF2BP2 may also have a regulatory role in EMT and cell cycle signaling. These processes are crucial for maintaining the proliferative and invasive properties of HNSCC cells. In vitro experiments have supported the role of IGF2BP2 in promoting the proliferation, migration, and invasion of HNSCC cells. Moreover, an orthotopic xenograft model has further validated its contribution to HNSCC tumorigenesis and CLN metastasis. In conclusion, these findings shed light on the invasion and metastasis of HNSCC by identifying IGF2BP2 as an SE-associated gene.

Epigenetic characteristics linked to the assembly and functioning of SE involve increased levels of H3K27Ac modification, recruitment of BRD4, Mediator complex, RNA Pol II, CDK7-containing TFIIH, and CDK9-containing P-TEFb, as well as binding of CBP/p300 acetyltransferase [7]. BRD4, upon binding to the acetylated chromatin, summons the Mediator complex, RNA Pol II, TFIIH, and P-TEFb to support the initiation and elongation of transcription, ultimately leading to the activation of target genes [49, 50]. CDK7, a member of the CDK family, regulates RNA Pol II phosphorylation and governs transcription initiation, pausing, and elongation. It serves as a crucial element of the transcription complex, with a preference for binding to SEs, driving the expression of SE-associated genes [51]. Histone acetyltransferases CBP/p300 operate as transcriptional coactivators, inducing an increase in H3K27Ac levels in promoters, enhancers, and SEs of target genes. This instigates the assemblage of diverse transcription components, thereby initiating gene transcription [52]. In comparison to enhancers, SEs have heightened vulnerability to transcription-associated inhibitors. The interaction between the SE region and its corresponding transcription components can be specifically impeded through CRISPR/Cas9 interference or the usage of small molecule inhibitors that target SEs. This selective inhibition leads to the suppression of transcription and expression of SE-associated genes [36, 53]. In our investigation, we divided the highly enriched H3K27Ac SE region of IGF2BP2 into three segments (E1, E2, and E3). By implementing CRISPR/Cas9 technology, we targeted and edited E1, E2, and E3 to disrupt the interactions between the SE and the promoter. Our findings demonstrate that inhibiting these three independent segments results in the suppression of transcriptional expression of IGF2BP2. BRD4 and MED1 co-localize on acetylated chromatin, particularly H3K27, which consequently affects the transcriptional activity controlled by SEs [9]. In hepatocellular carcinoma, silencing BRD4 or MED1 repressed the transcription of SE-associated genes like SPHK1, E2F2, CCND1, MYCN, and MYC [36]. Similarly, silencing MED1 hindered the transcription of TP63, MET, BIRC, and MMP3, which are SE-associated genes in HNSCC cells. Additionally, silencing BRD4 or MED1 significantly decreased the enrichment of BRD4 or MED1 in TP63-SE and MET-SE [54]. Interestingly, the mRNA and protein levels of IGF2BP2 were also suppressed upon silencing BRD4 or MED1. ChIP-qPCR assays confirmed that silencing BRD4 or MED1 led to a decrease in the enrichment of BRD4 or MED1 on IGF2BP2-SE in HNSCC cells, respectively, resulting in the inhibition of IGF2BP2 transcriptional expression. Furthermore, treatment of HNSCC cells with small molecule inhibitors THZ1, JQ1, OTX-015, and CPI-637 specifically reduced the mRNA and protein levels of IGF2BP2 by inhibiting the SE-associated transcriptional program. Subsequent ChIP-qPCR analysis also verified that JQ1 or CPI-637 treatment significantly reduced the enrichment of BRD4 or H3K27Ac at IGF2BP2-SE in HNSCC cells, respectively. These findings support the notion that IGF2BP2-SE exerts a positive regulatory role in maintaining the functional characteristics of HNSCC by governing the transcriptional expression of IGF2BP2.

TFs are proteins that regulate gene transcription by forming transcriptional complexes with RNA Pol II and binding to specific DNA sequences of target genes. Depending on specific spatial and temporal conditions, TFs can either activate or repress gene transcription [55]. TFs bind to oncogenic SEs associated with specific signaling pathways and enhance the transcriptional activity of oncogenes, thus promoting tumor development and progression [56]. In our correlation analysis, using the JASPAR database prediction, as well as TCGA and GEO data, we found that KLF7 exhibited the strongest correlation with the expression of IGF2BP2. Furthermore, we have demonstrated that KLF7 promotes the transcriptional expression of IGF2BP2 by binding to its SE and promoter regions. KLF7 belongs to the Krüppel family of transcriptional regulators and is located on chromosome 2q33.3. It functions as a transcriptional activator [57]. Notably, it is highly expressed in high-grade plasma ovarian cancer, pancreatic ductal adenocarcinoma, hepatocellular carcinoma, and breast cancer. Additionally, its expression is associated with clinical stage, pathological grade, and metastasis in these cancers [58–61]. Similarly, our analysis of comprehensive public databases and clinical samples revealed that KLF7 is highly expressed in HNSCC and strongly correlates with malignant progression and poor prognosis in patients. Several cohorts also identified a significant correlation between KLF7 and IGF2BP2 mRNA and protein expression in HNSCC, highlighting a robust and compelling relationship. Finally, our findings suggest that high expression levels of KLF7 and IGF2BP2 are associated with shorter OS and DFS, underscoring the critical role of the KLF7/IGF2BP2 axis in promoting malignant progression in HNSCC.

Studies have provided evidence that blocking SE-associated transcriptional programs can disrupt oncogene transcription and hinder tumor growth, presenting a unique approach to treating cancer [62–65]. Notably, the SE-associated transcriptional regulatory process involves various components, such as BRD4, CDK7, CDK9, and CBP/p300, which are potential targets for small molecule inhibitors. Numerous small molecule inhibitors targeting the SE-associated transcriptional program have been assessed in preclinical models and clinical trials, demonstrating promising activity against different types of advanced cancers [20, 66, 67]. In line with these discoveries, our investigation shows that JQ1 treatment effectively suppresses the proliferation and invasive metastatic ability of HNSCC cells in both in vitro and in vivo experiments. While BRD4, CDK7, CDK9, and CBP/p300 are commonly found binding proteins on transcriptional regulatory elements, they are highly enriched in oncogenic SEs. Therefore, their inhibitory effects primarily impact the transcription of SE-associated oncogenes, which ultimately leads to the attenuation of the malignant characteristics in cancer cells. In the future, further exploration into the specific elements and underlying mechanisms of SE-associated oncogenes will aid in the identification of novel therapeutic targets and the development of more precise and potent cancer therapies.

## Conclusions

In summary, our study reveals the clinical and biological functions of IGF2BP2 as a SE-associated gene in promoting the malignant progression of HNSCC. In terms of mechanism, we have observed that the expression of IGF2BP2, driven by SE, undergoes transcriptional activation through the involvement of KLF7, as well as other transcriptional elements such as BRD4 and MED1. These elements play a role in the abnormal addiction to transcription of IGF2BP2. Furthermore, we have provided evidence to support the notion that targeting SE-driven transcriptional programs could serve as an effective strategy for intervening therapeutically. These discoveries have the potential to offer valuable clinical evidence for the exploration of an innovative prognostic biomarker and treatment approach aimed at eradicating HNSCC.

### Supplementary Information

**Supplementary Material 1.**

**Supplementary Material 2.**

**Supplementary Material 3.**

**Supplementary Material 4.**

**Supplementary Material 5.**

**Supplementary Material 6.**

**Supplementary Material 7.**

## Data Availability

The data supporting the conclusions of this article are included within the article and its additional files.

## Abbreviations

HNSCC: Head and neck squamous cell carcinoma
CLN: Cervical lymph node
SE: Super-enhancer
TCGA: The Cancer Genome Atlas
GEO: Gene Expression Omnibus
ChIP-seq: Chromatin immunoprecipitation-sequencing
H3K27Ac: Histone 3 lysine 27 acetylation
H3K4me1: Histone 3 lysine 4 monomethylation
Hi-C: High-throughput Chromosome Conformation Capture
WGCNA: Weighted gene coexpression network analysis
IGF2BP2: Insulin-like growth factor 2 mRNA-binding protein 2
TF: Transcription factor
BRD4: Bromodomain-containing protein 4
MED1: Mediator complex subunit 1
RNA Pol II: RNA polymerase II
ROSE: Rank Ordering of Super-Enhancers
CDK7: Cyclin-dependent kinases 7
P-TEFb: Positive transcription elongation factor b
KLF7: Krueppel-Like Factor 7
GSEA: Gene set enrichment analysis
KEGG: Kyoto Encyclopedia of Genes and Genomes
ANCT: Adjuvant non-cancerous tissues
SUSY: Sun Yat-sen University
OS: Overall survival
DFS: Disease-free survival
IHC: Immunohistochemistry
DAB: Diaminobenzidine
FBS: Fetal bovine serum
shRNA: Short hairpin RNA
sgRNA: Small guide RNA
siRNA: Small interfering RNA
SDS-PAGE: Sodium dodecyl sulfate–polyacrylamide gel electrophoresis
PVDF: Polyvinylidene fluoride
qRT-PCR: Quantitative real-time PCR
CCK-8: Cell counting kit-8
ORF: Open reading frame
H&E: Hematoxylin-eosin
WT: Wild-type
EMT: Epithelial-mesenchymal transition
m^6^A: N6-methyladenosine
TAD: Topologically associated domain
