# Supplementary material for: KLF7 regulates super-enhancer-driven IGF2BP2 overexpression to promote the progression of head and neck squamous cell carcinoma
Source: J Exp Clin Cancer Res. 2024 Mar 5;43:69. doi: 10.1186/s13046-024-02996-y (PMC10913600; doi:10.1186/s13046-024-02996-y)
Supplement: Supplementary file 1 — Supplementary Material 1. [file 13046_2024_2996_MOESM1_ESM.docx]

**Table S1. The sequences of the shRNA, sgRNA, and siRNA** **oligonucleotides.**

| **Gene names** | **Sequences (5’-3’)** |
| --- | --- |
| shIGF2BP2-1 | ACCGGTGGCAGATGAGACCAAACTATTCAAGAGATAGTTTGGTCTCATCTGCCTTTTTTGAATTC |
| shIGF2BP2-2 | ACCGGTGAAGTGATCGTCAGAATTATTCAAGAGATAATTCTGACGATCACTTCTTTTTTGAATTC |
| IGF2BP2-SE1-sgRNA | GAACTTTCTCAACTTGATGA |
| IGF2BP2-SE2-sgRNA | GGACCTCCGCATTTCTTTGT |
| IGF2BP2-SE3-sgRNA | GGCATGAAAGAAGTGAAACC |
| siBRD4-1 | Sense: CCUCCCUGAUUACUAUAAGAUTT  Antisense: AUCUUAUAGUAAUCAGGGAGGTT |
| siBRD4-2 | Sense: CAGUGACAGUUCGACUGAUGATT  Antisense: UCAUCAGUCGAACUGUCACUGTT |
| siMED1-1 | Sense: GCCGAGUUCCUCUUAUCCUAATT  Antisense: UUAGGAUAAGAGGAACUCGGCTT |
| siMED1-2 | Sense: GCAGUAAAUCAGAAGGUUCAUTT  Antisense: AUGAACCUUCUGAUUUACUGCTT |
| siKLF7-1 | Sense: GGAGGAUCUCAGAGACCUUUGTT  Antisense: CAAAGGUCUCUGAGAUCCUCCTT |
| siKLF7-2 | Sense: GCUCUUCUCUAGACAGCUACATT  Antisense: UGUAGCUGUCUAGAGAAGAGCTT |

**Abbreviations:** shRNA: short hairpin RNA; sgRNA: small guide RNA; siRNA: small interfering RNA; SE: super-enhancer.

**Table S2.** **Primer sequences.**

| **Gene names** | **Sequences (5’-3’)** | **Application** |
| --- | --- | --- |
| IGF2BP2 | Forward: AGTGGAATTGCATGGGAAAATCA | qRT-PCR |
|  | Reverse: CAACGGCGGTTTCTGTGTC |  |
| β-ACTIN | Forward: CTACCTCATGAAGATCCTCACCGA | qRT-PCR |
|  | Reverse: TTCTCCTTAATGTCACGCACGATT |  |
| BRD4 | Forward: GAGCTACCCACAGAAGAAACC | qRT-PCR |
|  | Reverse: GAGTCGATGCTTGAGTTGTGTT |  |
| MED1 | Forward: GAAGTGTTGGCTATCTCACACC | qRT-PCR |
|  | Reverse: TGTCATCCAGTAGGTCAGAAGG |  |
| KLF7 | Forward: AGACATGCCTTGAATTGGAACG  Reverse: GGGGTCTAAGCGACGGAAG | qRT-PCR |
| IGF2BP2-Promoter | Forward: GGAGGAAACAACGTGACCGA  Reverse: GGGACCAGGCTCTAACCATC | ChIP-qPCR |
| IGF2BP2-SE | Forward: CCCACCTCTCCATTCCGCTA  Reverse: TTCGGCTCGTTTTCTCGGG | ChIP-qPCR |
| IGF2BP2-NEG | Forward: AGCACACAAAAGAGGAGGGAAA  Reverse: AGAGTCCCTTAGGAGTGCTTAC | ChIP-qPCR |

**Abbreviations:** SE: super-enhancer; NEG: negative; qRT-PCR: quantitative real-time PCR; ChIP-qPCR: chromatin immunoprecipitation qPCR.

**Table S3. Correlation of IGF2BP2 expression with potential transcription factors in HNSCC datasets**

| **transcription factors** | ***R-value***  **TCGA** | ***P-*value**  **TCGA** | ***R-value***  **GSE30784** | ***P-*value**  **GSE30784** | ***R-*value**  **GSE42743** | ***P-value***  **GSE42743** | ***R-value***  **GSE41613** | ***P-value***  **GSE41613** |
| --- | --- | --- | --- | --- | --- | --- | --- | --- |
| KLF7 | 0.481 | 1.95E-30 | 0.457 | 5.52E-10 | 0.623 | 3.19E-09 | 0.466 | 1.49E-06 |
| TFAP2A | 0.229 | 2.10E-07 | 0.178 | 0.021509 | 0.508 | 3.87E-06 | 0.221 | 0.029724 |
| KLF10 | 0.326 | 6.42E-14 | 0.359 | 1.95E-06 | 0.429 | 0.000136 | 0.199 | 0.050626 |
| TFAP2C | 0.227 | 2.90E-07 | 0.17 | 0.027936 | 0.377 | 0.000935 | -0.06 | 0.562536 |
| SP1 | 0.139 | 0.001739 | -0.36 | 2.46E-06 | 0.296 | 0.010399 | 0.187 | 0.066266 |
| SP2 | 0.299 | 7.75E-12 | 0.039 | 0.614152 | 0.284 | 0.014076 | 0.078 | 0.448066 |
| KLF4 | 0.025 | 0.581525 | 0.031 | 0.689217 | 0.195 | 0.095615 | 0.147 | 0.151403 |
| MAZ | 0.215 | 1.17E-06 | 0.12 | 0.123822 | 0.156 | 0.184379 | 0.312 | 0.001885 |
| ZNF530 | 0.061 | 0.174191 | -0.01 | 0.88486 | 0.115 | 0.327681 | 0.111 | 0.278589 |
| ZNF148 | 0.268 | 1.08E-09 | 0.114 | 0.143673 | 0.099 | 0.402419 | 0.203 | 0.046138 |
| ZNF257 | -0.02 | 0.600976 | -0.06 | 0.461922 | 0.027 | 0.819147 | 0.022 | 0.828671 |
| SP4 | 0.088 | 0.049707 | -0.21 | 0.006182 | 0.002 | 0.98745 | 0.087 | 0.39468 |
| ZNF417 | 0.101 | 0.023068 | -0 | 0.967315 | -0.02 | 0.862665 | -0.1 | 0.319541 |
| KLF1 | -0.33 | 3.08E-14 | 0.03 | 0.702141 | -0.14 | 0.228616 | 0.078 | 0.450395 |
| ZKSCAN5 | 0.237 | 7.29E-08 | 0.243 | 0.001583 | -0.16 | 0.173139 | 0.112 | 0.276508 |
| KLF12 | 0.12 | 0.007068 | -0.31 | 4.42E-05 | -0.16 | 0.164632 | -0.15 | 0.144651 |
| KLF9 | 0.155 | 0.000494 | 0.069 | 0.372254 | -0.18 | 0.130723 | 0.131 | 0.200519 |
| KLF14 | 0.269 | 8.85E-10 | -0.07 | 0.348978 | -0.2 | 0.081067 | 0.127 | 0.216545 |
| WT1 | 0.024 | 0.585165 | 0.132 | 0.087944 | -0.26 | 0.023712 | 0.016 | 0.872993 |
| ZNF460 | 0.226 | 3.18E-07 | -0.06 | 0.427889 | -0.27 | 0.019687 | -0.03 | 0.752682 |
| EGR1 | 0.101 | 0.023377 | -0.05 | 0.53898 | -0.28 | 0.01653 | -0.001 | 0.994912 |
| PRDM9 | 0.004 | 0.91994 | -0.07 | 0.357126 | -0.32 | 0.005493 | 0.075 | 0.467871 |
| SP5 | -0.35 | 2.64E-16 | -0.41 | 4.56E-08 | -0.32 | 0.005203 | -0.29 | 0.004471 |
| PATZ1 | 0.029 | 0.509975 | -0.28 | 0.000297 | -0.32 | 0.005018 | -0.05 | 0.611609 |
| ZFP14 | -0.13 | 0.004417 | -0.03 | 0.70389 | -0.33 | 0.004665 | -0.08 | 0.45644 |
| ZNF610 | 0.154 | 0.000536 | 0.035 | 0.649157 | -0.46 | 3.36E-05 | -0.17 | 0.090871 |
| KLF15 | -0.09 | 0.044495 | -0.11 | 0.170423 | -0.51 | 4.03E-06 | 0.076 | 0.458576 |
| ZNF263 | 0.185 | 2.91E-05 |  |  |  |  |  |  |
| ZNF454 | -0.14 | 2.02E-03 |  |  |  |  |  |  |
