# Supplementary material for: KLF7 regulates super-enhancer-driven IGF2BP2 overexpression to promote the progression of head and neck squamous cell carcinoma
Source: J Exp Clin Cancer Res. 2024 Mar 5;43:69. doi: 10.1186/s13046-024-02996-y (PMC10913600; doi:10.1186/s13046-024-02996-y)
Supplement: Supplementary file 4 — Supplementary Material 4. [file 13046_2024_2996_MOESM4_ESM.docx]

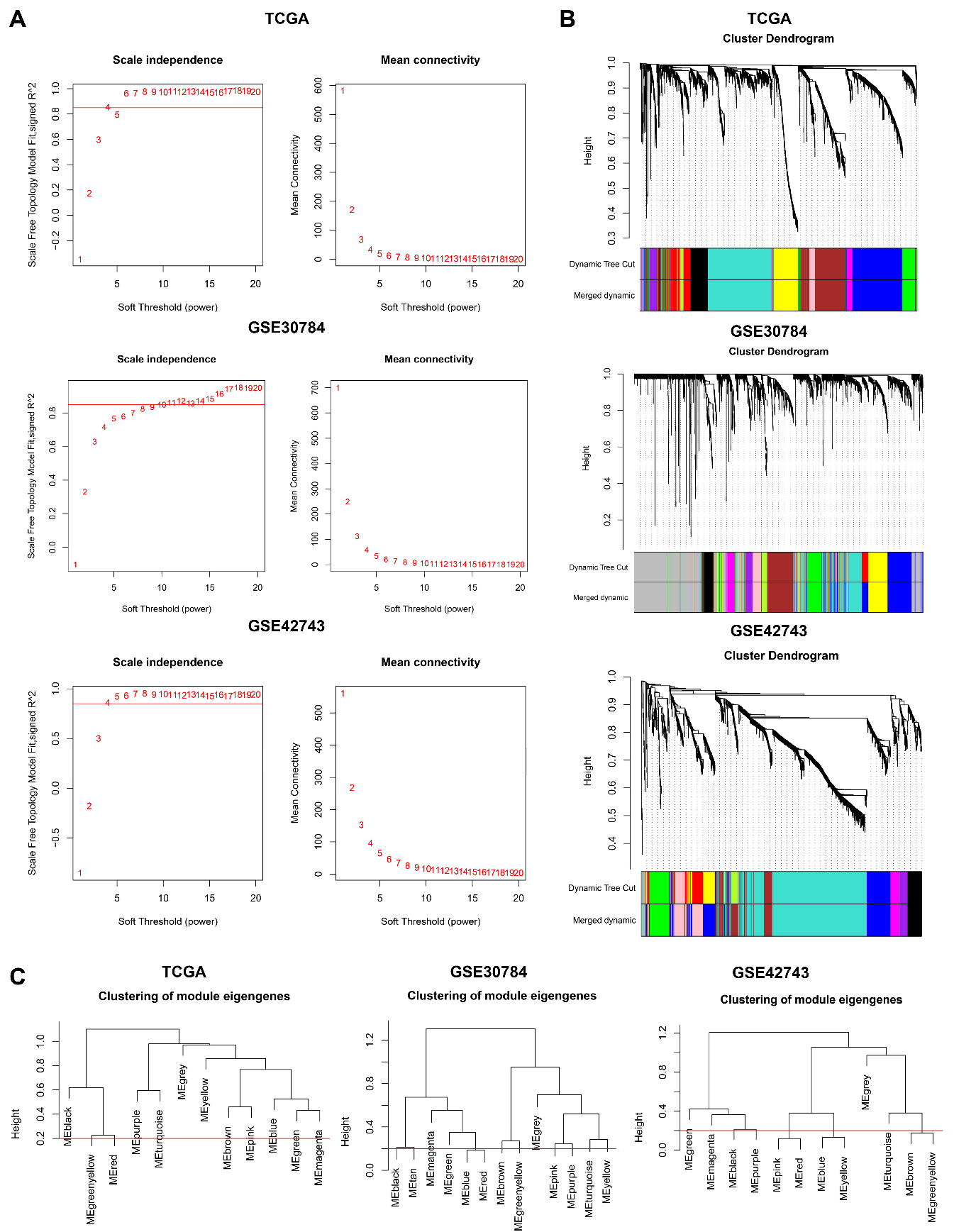


**Fig S1. Screening of key modules associated with HNSCC using WGCNA**. **A** Analysis of scale-free gene network topology and mean connectivity across soft-threshold powers in the TCGA-HNSCC, GSE30784, and GSE42743 datasets cohort. **B** Dendrogram showing the differential gene clustering based on topological overlap in the TCGA-HNSCC, GSE30784, and GSE42743 datasets. **C** Clustering of consensus module eigengenes for the TCGA-HNSCC, GSE30784, and GSE42743 datasets, where genes with correlations between modules > 0.8 below the red line were merged.


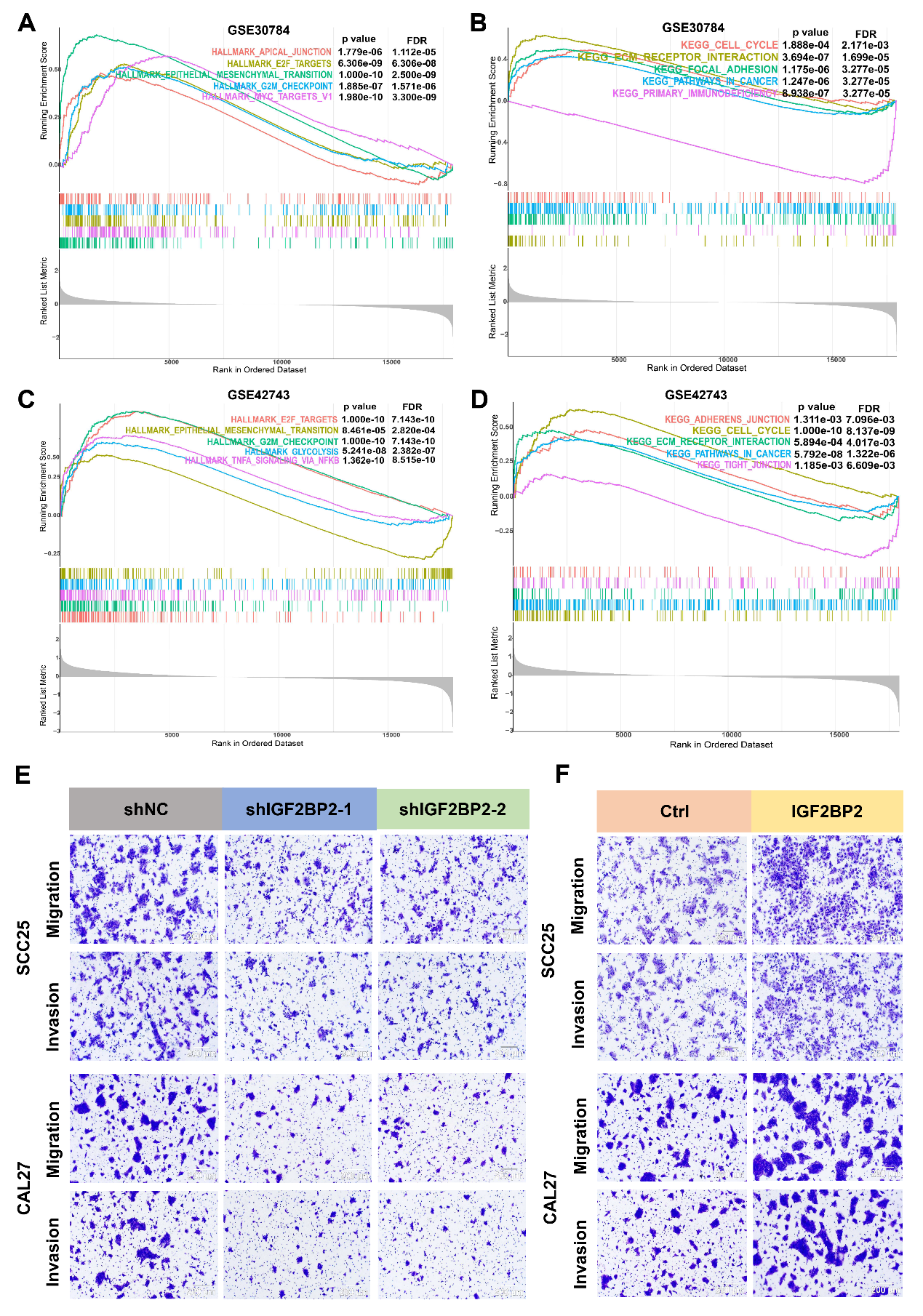


**Fig S2. Functional enrichment analysis of IGF2BP2 in HNSCC. A** GSEA results showing functional enrichment of IGF2BP2 expression in Hallmark gene sets in the GSE30784 dataset. **B** GSEA results show functional enrichment of IGF2BP2 expression in KEGG gene sets in the GSE30784 dataset. **C** GSEA results showing functional enrichment of IGF2BP2 expression in Hallmark gene sets in the GSE42743 dataset. **D** GSEA results show functional enrichment of IGF2BP2 expression in KEGG gene sets in the GSE42743 dataset.


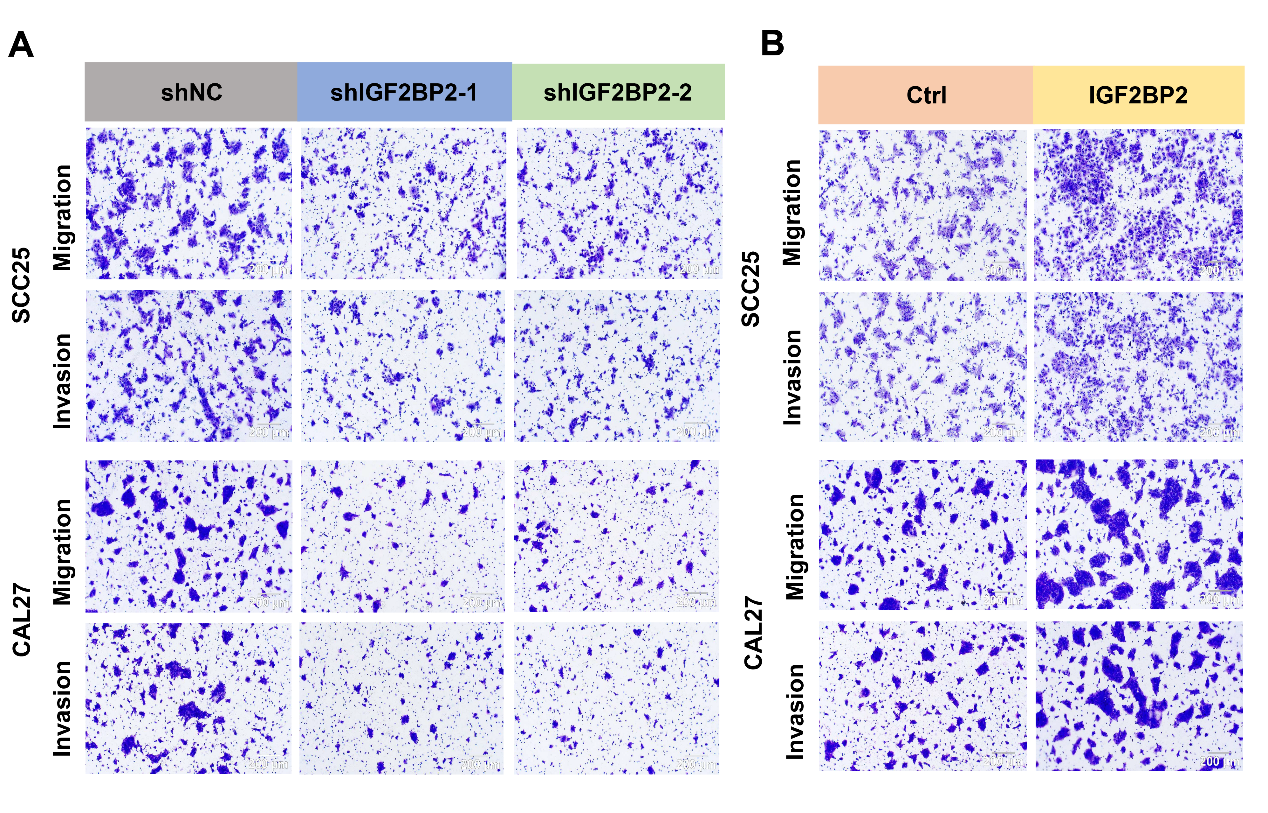


**Fig S3. IGF2BP2 promotes the migration and invasion of HNSCC cells. A** The migration and invasion of IGF2BP2 knockdown SCC25 and CAL27 cells were photographed. Scale bar, 200μm. **B** The migration and invasion of IGF2BP2 overexpression SCC25 and CAL27 cells were photographed. Scale bar, 200μm.


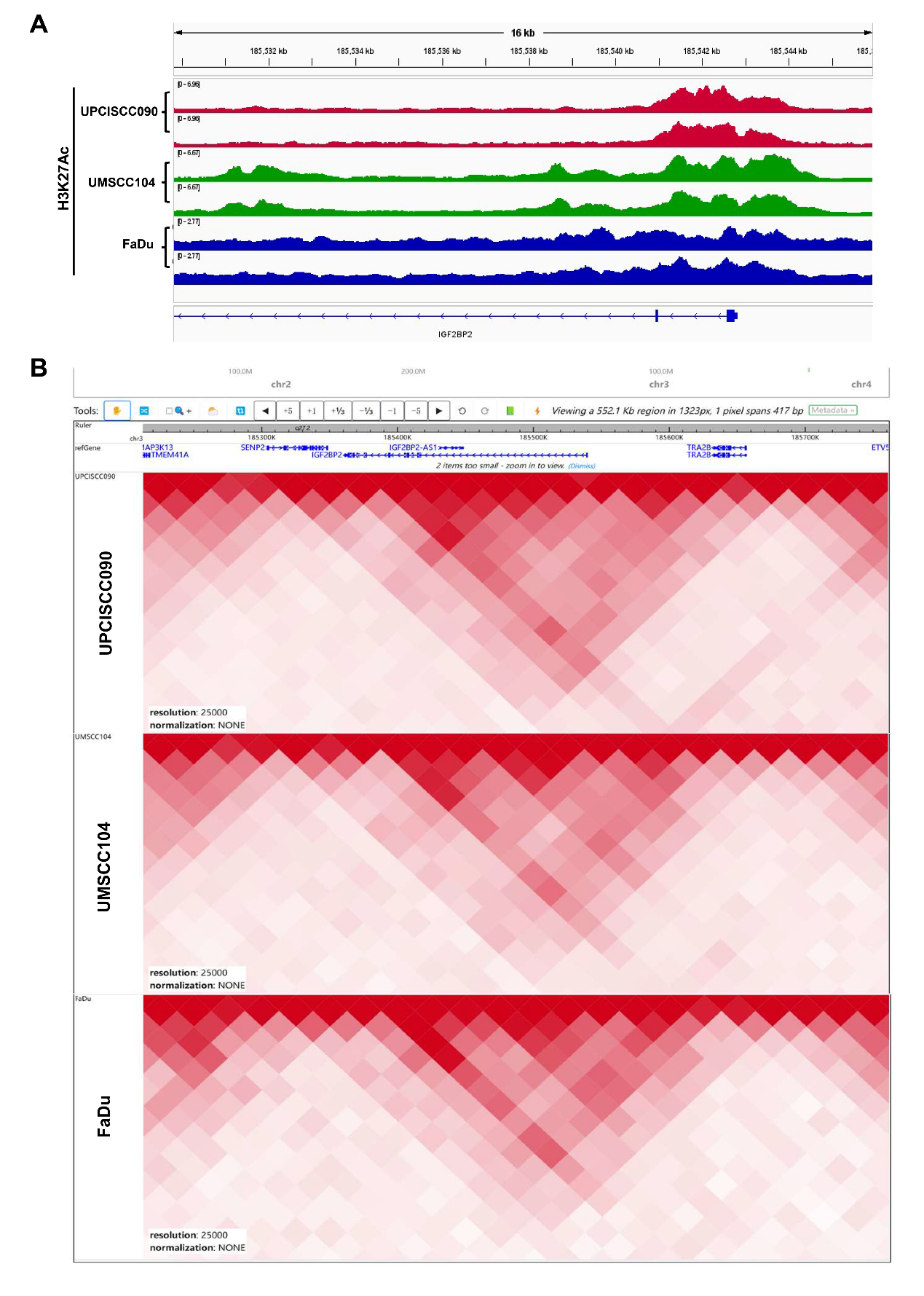


**Fig S4. H3K27Ac signal and Hi-C visualization of UPCI-SCC-090, UM-SCC-104, and FaDu cells support** **the looping of SE element with IGF2BP2 promoter. A** Visualization of H3K27Ac level of IGF2BP2 with IGV Genome Browser. **B** IGF2BP2 in UPCI-SCC-090, UM-SCC-104, and FaDu cells topologically associated domain (TAD) region was predicted based on the Hi-C data.
